# Supplementary material for: The GABARAP Co-Secretome Identified by APEX2-GABARAP Proximity Labelling of Extracellular Vesicles
Source: Cells. 2020 Jun 16;9(6):1468. doi: 10.3390/cells9061468 (PMC7349886; doi:10.3390/cells9061468)
Supplement: Supplementary file 1 [file cells-09-01468-s001.zip › Supplementary data/APEX2-GABARAP_Supplemental_Figures.docx]

# Supplementary Figures

**The GABARAP co-secretome identified by APEX2-GABARAP proximity labelling of extracellular vesicles**

Julia L. Sanwald ^1,2^, Gereon Poschmann ^3^, Kai Stühler ^3,4^, Christian Behrends ^5^, Silke Hoffmann ^2^, Dieter Willbold ^1,2^

^1^ Institut für Physikalische Biologie, Heinrich-Heine-Universität Düsseldorf, Universitätsstraße 1, 40225 Düsseldorf, Germany

^2^ Institute of Biological Information Processing (IBI-7: Structural Biochemistry), Forschungszentrum Jülich, Leo-Brandt-Straße, 52428 Jülich, Germany

^3^ Institute of Molecular Medicine I, Heinrich-Heine-Universität Düsseldorf, Universitätsstraße 1, 40225 Düsseldorf, Germany

^4^ Molecular Proteomics Laboratory, Biologisch-Medizinisches Forschungszentrum (BMFZ), Heinrich-Heine-Universität Düsseldorf, Universitätsstraße 1, 40225 Düsseldorf, Germany

^5^ Munich Cluster for Systems Neurology (SyNergy), Ludwig‐Maximilians‐Universität München, Feodor-Lynen-Straße 17, 81377 München, Germany

Supplementary Figure S1 is related to Introduction

Supplementary Figure S2 is related to Figure 1A

Supplementary Figure S3 is related to Figure 1

Supplementary Figure S4 is related to Figure 1

Supplementary Figure S5 is related to Figure 2

Supplementary Figure S6 is related to Figure 2B and 2D

Supplementary Figure S7 is related to Figure 3B-3C

Supplementary Figure S8 is related to Figure 4A

Supplementary Figure S9 is related to Figure 4B-4E

Supplementary Figure S10 is related to Figure 5A

Supplementary Figure S11 is related to Discussion

## Supplementary Figure S1

##
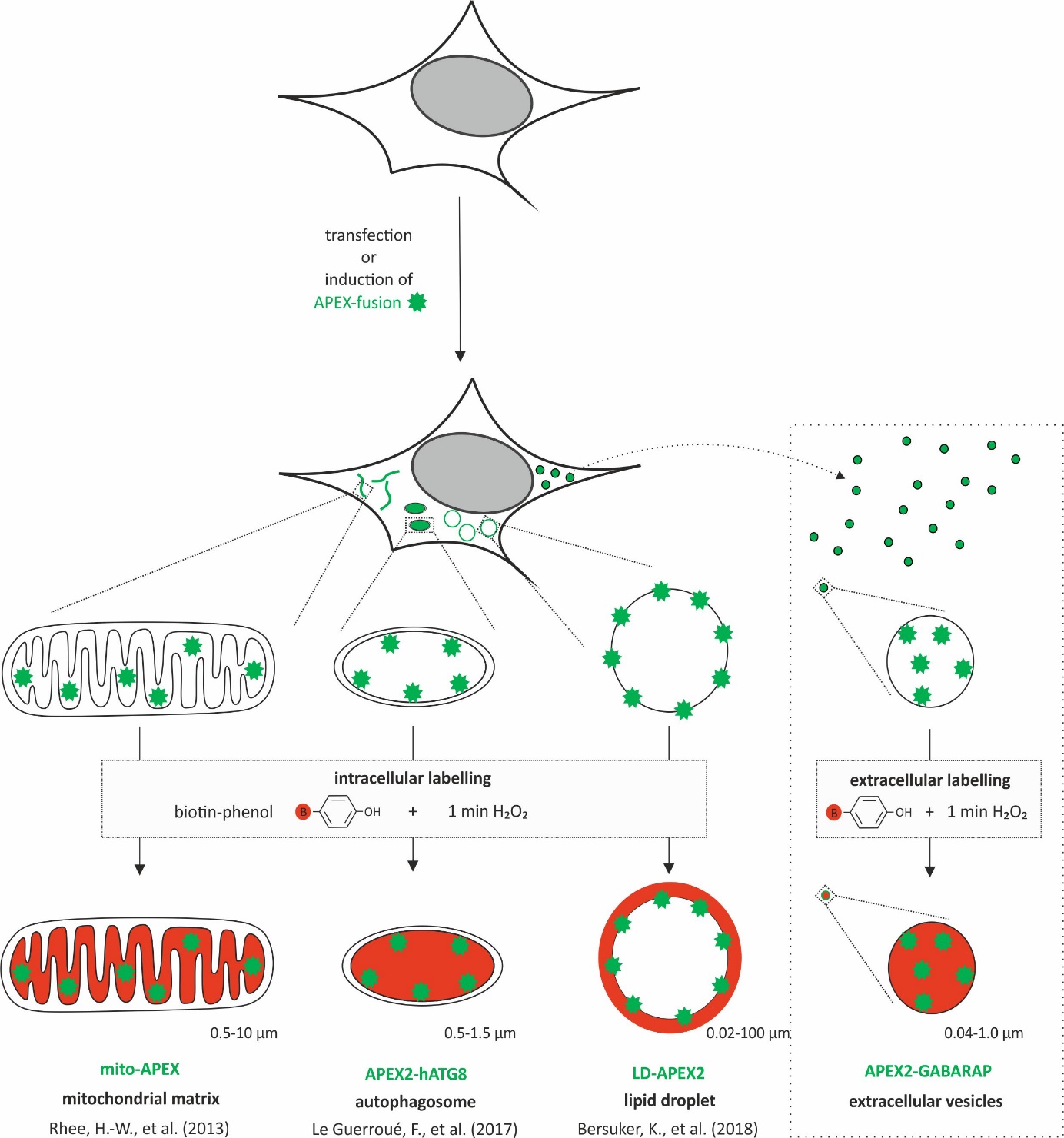


Supplementary Figure S1. APEX2-mediated proximity labelling in intra- and extracellular compartments of mammalian cells. Schematic of cellular compartments in which APEX2-mediated proximity labelling was applied previously (from leftmost to second from right): Mitochondrial matrix, autophagosome, and lipid droplets. For these compartments, labelling was conducted intracellularly. Here, we demonstrate the method’s applicability for extracellular compartments, such as extracellular vesicles (rightmost). This schematic does not represent the actual size dimensions and was upscaled for enhanced visibility.

## Supplementary Figure S2


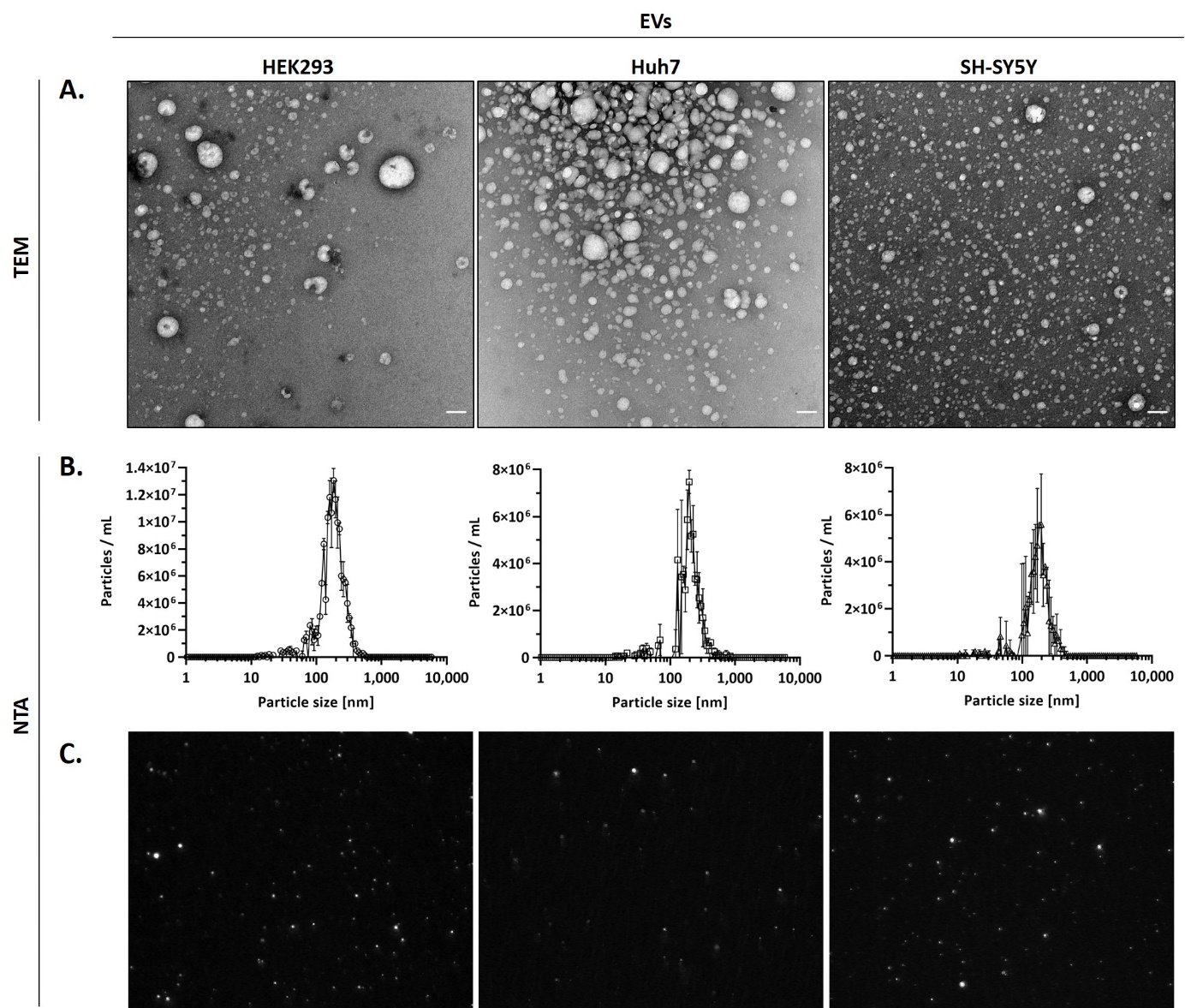


**Supplementary Figure S2.** Enrichment of EV samples from HEK293, Huh7, and SH-SY5Y cells was tested by TEM and NTA. (A) After ultracentrifugation-based isolation from cell culture supernatants, the EVs were resuspended in 2 % PFA, contrasted in 4 % uranyl acetate, embedded in 10 % trehalose on formvar/carbon coated EM grids, and visualised by TEM. One representative image of at least five technical replicates is shown. Scale bar 100 nm. (B) Nanoparticle tracking analysis (NTA) of the EV samples shown under A. Concentrated cell culture media were subjected to NTA, resulting in a distribution of particle sizes ranging from 10.1 nm to 838.3 nm. Of each sample, two trackings were recorded. Particle counts and standard deviations were calculated after background subtraction. (C) Single frames of NTA measurement videos depicting the particle distribution. Representative data of two technical replicates are shown in (B) and (C).

## Supplementary Figure S3

##
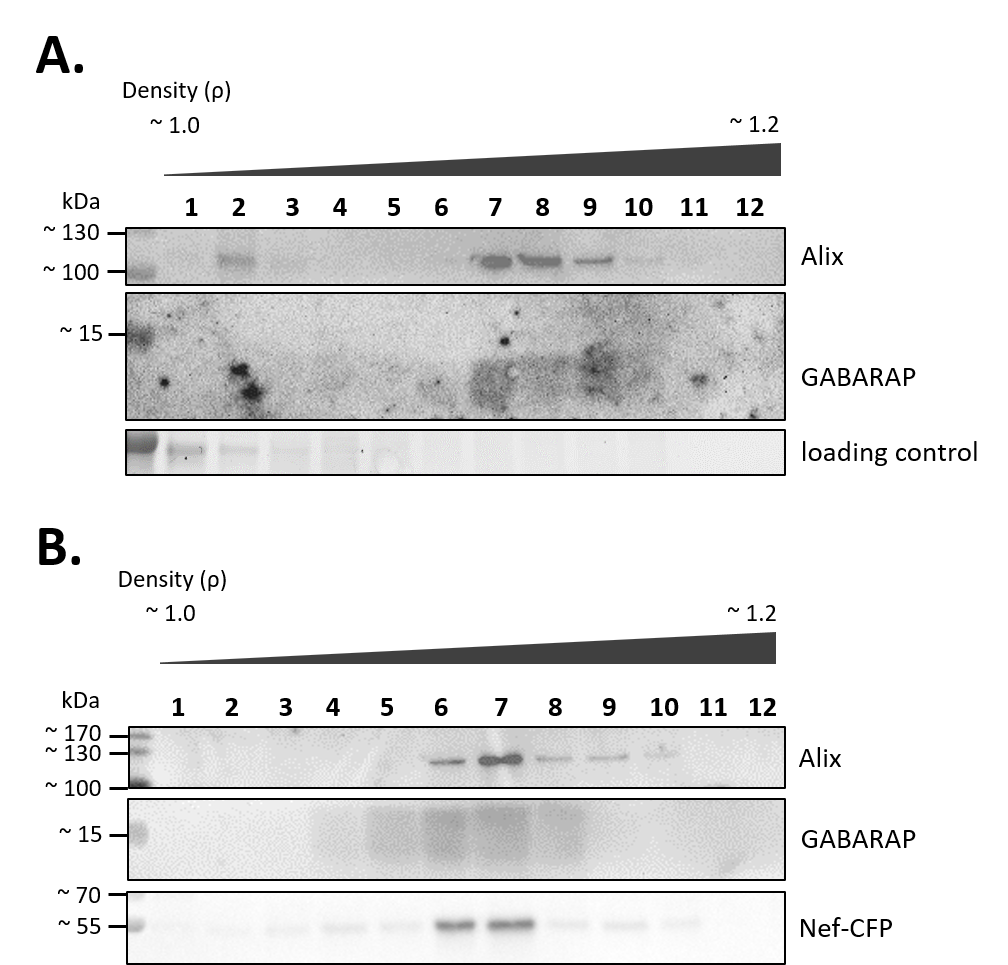


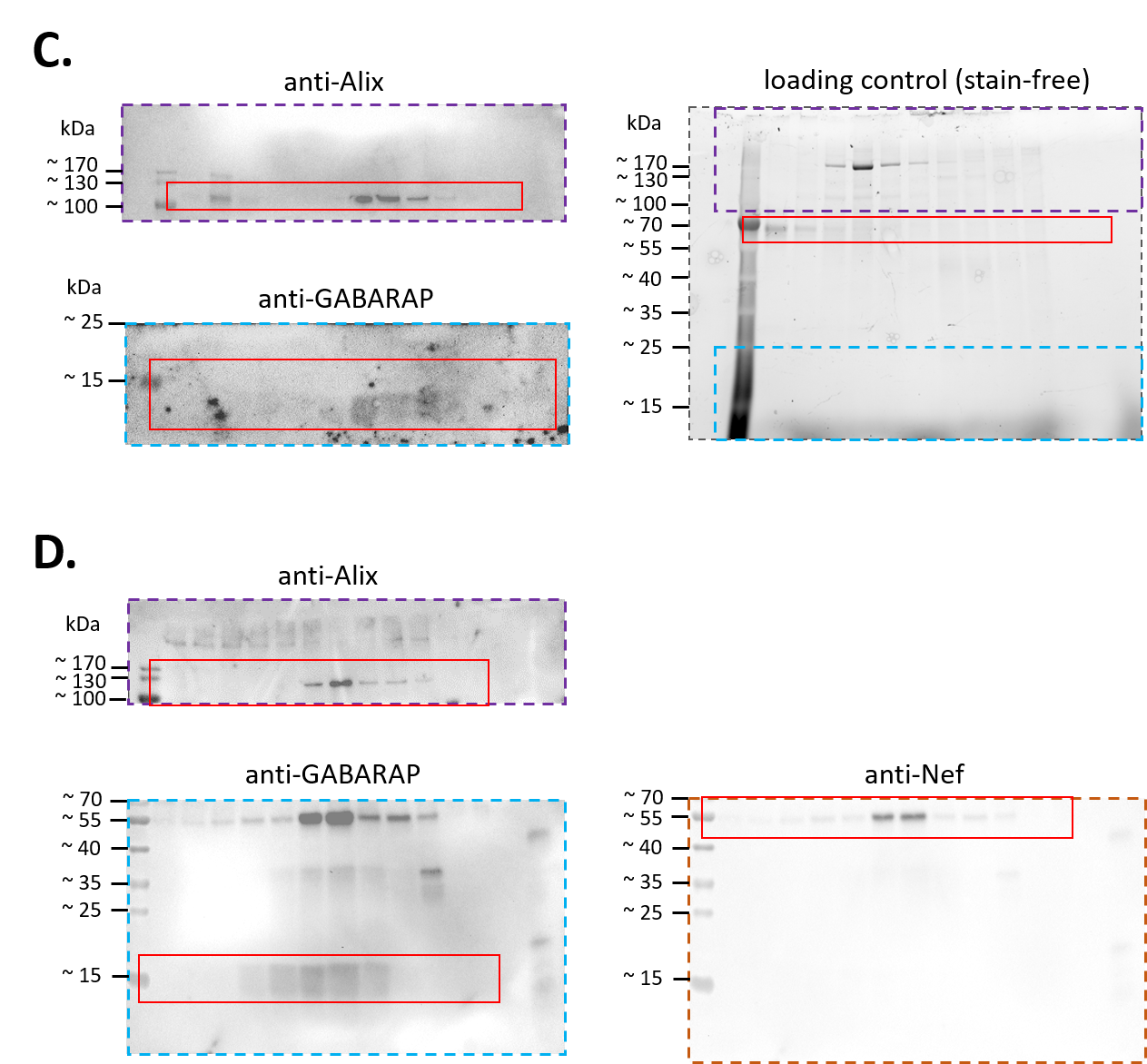


Supplementary Figure S3. GABARAP is detected in a density gradient. GABARAP is detected in density gradient fractions corresponding to the buoyant density of EVs. EVs were isolated (A) from HEK293 WT cells and (B) from HEK293 cells stably overexpressing HIV-1 Nef, a protein known to trigger both its own and EV secretion in general. One representative blot out of three, each performed with cell lysates from different passage numbers, is shown. Before EV isolation by ultracentrifugation, larger vesicles were removed by centrifugation at 10,000 x g. A representative crop of a stain-free gel is shown as loading control. (C) Uncropped immunoblots related to (A). The cropped region is indicated by red box. (D) Uncropped immunoblots related to (B). The cropped region is indicated by red box. Dashed coloured boxes in the stain-free image correspond to the respective areas into which the blot was cut for incubation with different antibodies.

## Supplementary Figure S4


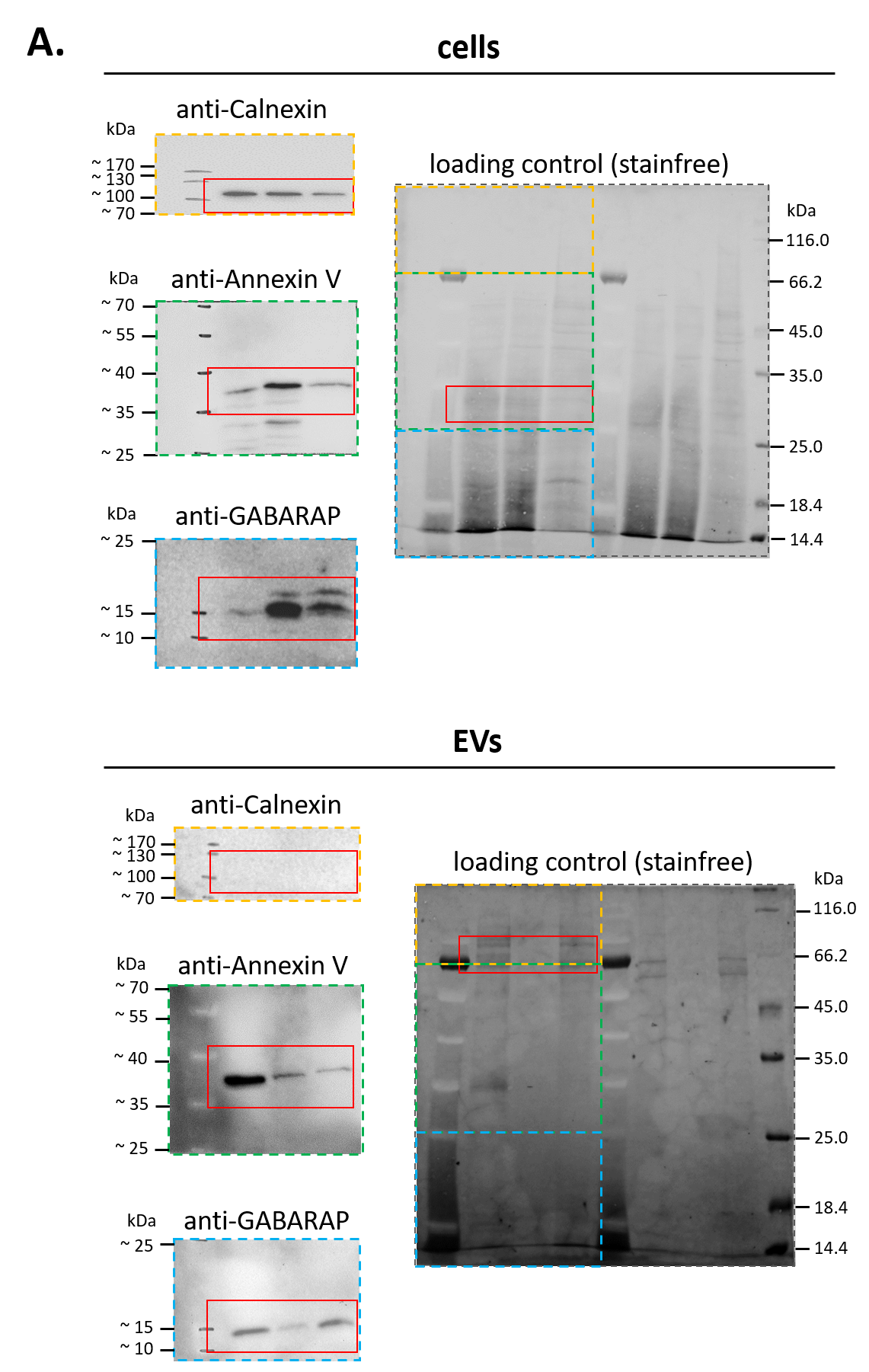


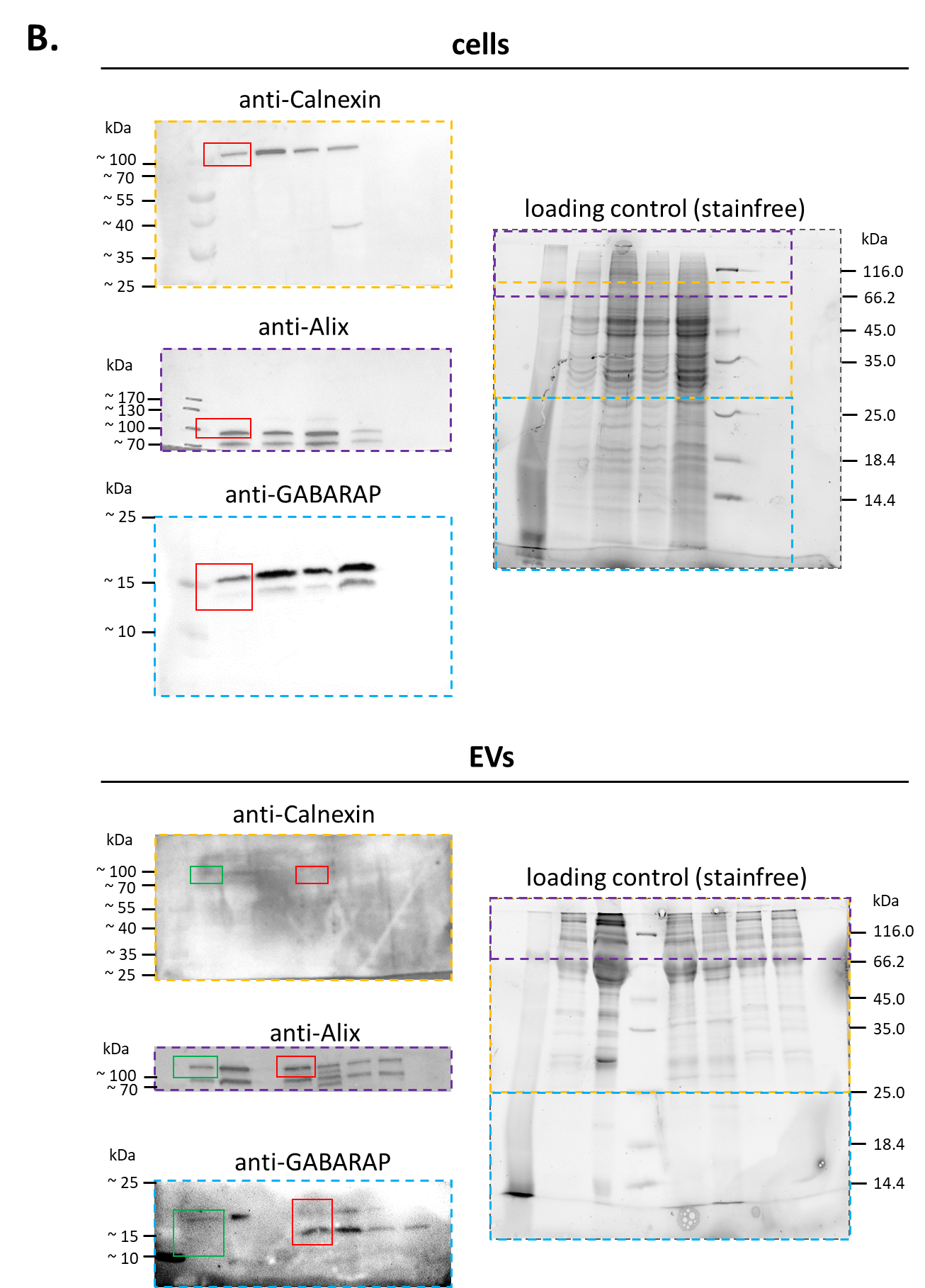


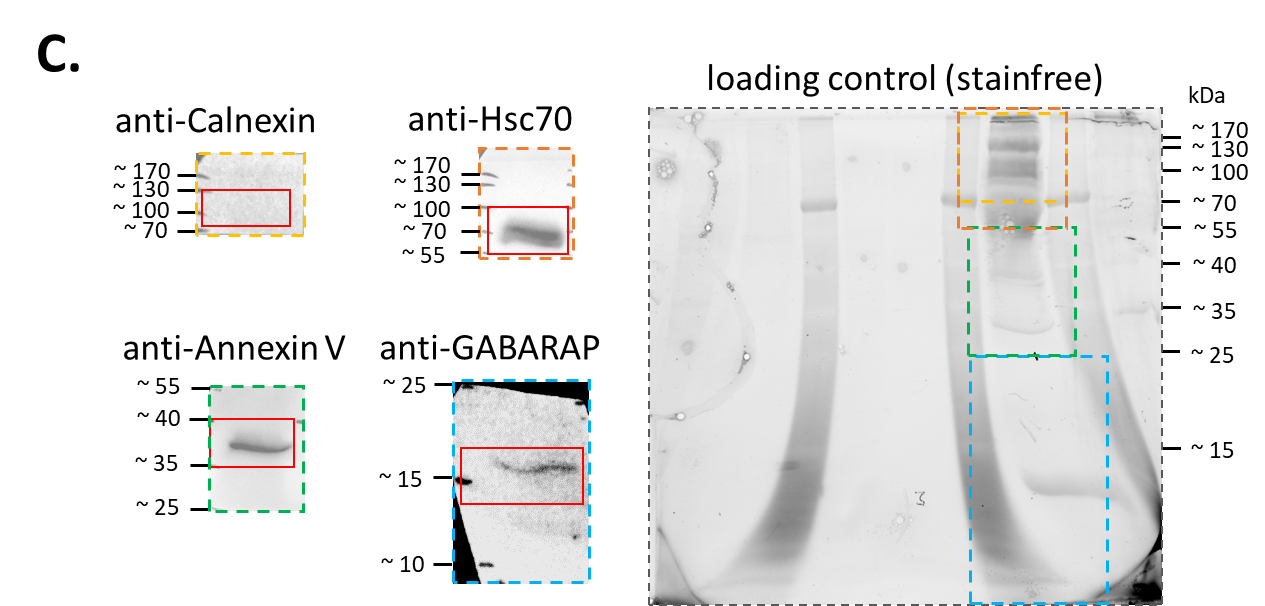


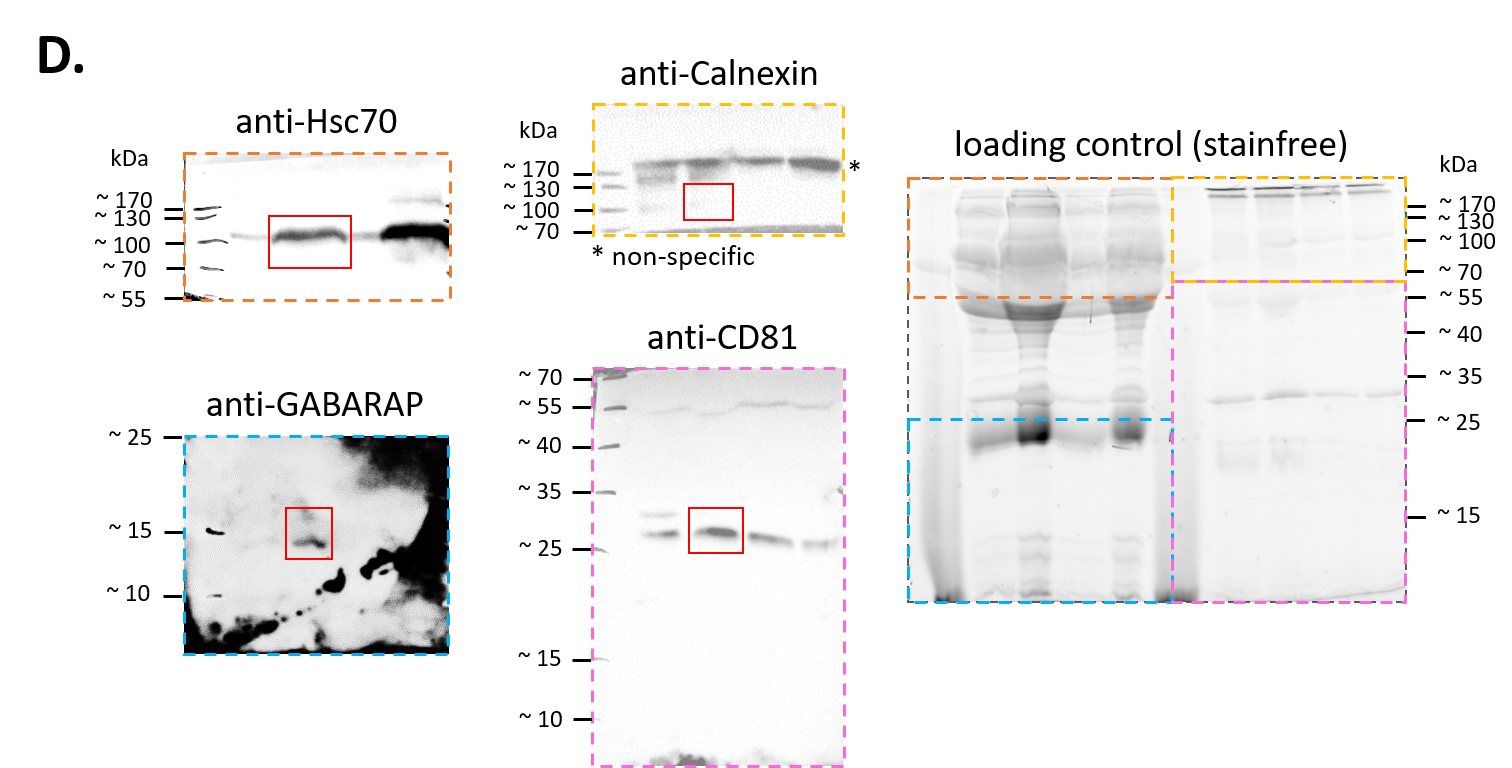


**Supplementary Figure S4.** Full images of blots shown in Figure 1A-1D. The cropped region is indicated by red box. GABARAP (dashed blue box) is secreted in EVs and is detectable by immunoblotting. Alix (dashed purple box), Annexin V (dashed green box), Hsc70 (dashed orange box), and CD81 (dashed magenta box) were used as EV marker proteins, and Calnexin (dashed yellow box) as marker for cellular impurities in an EV sample. (A) Uncropped versions of the blots and the corresponding stain-free gels related to Figure 1A. GABARAP is detected in EVs from the three human cell lines HEK293, Huh7, and SH-SY5Y after starvation. One representative blot of two different cellular passages is shown. EVs were obtained by ultracentrifugation. (B) Uncropped versions of the blots and the corresponding stain-free gels related to Figure 1B. Under fed conditions, predominantly lipidated GABARAP is detectable in HEK293 EVs, while unlipidated GABARAP was detected in unconditioned media (UCM; the cropped regions are indicated by green boxes). One representative blot of two different cellular passages is shown. EVs were prepared using polymer-based precipitation. (C) Uncropped versions of the blots and the corresponding stain-free gel related to Figure 1C. Unlipidated GABARAP is detectable in EVs isolated from bovine blood serum. One representative blot of two analysed EV batches, independently obtained from the same batch of FCS (Cat. No. F9665, Sigma-Aldrich), is shown. EVs were obtained by ultracentrifugation. (D) Uncropped versions of the blots and the corresponding stain-free gel related to Figure 1D. GABARAP is detectable in EVs isolated from human blood plasma. One representative blot of two analysed EV batches is shown. Respective EV samples were obtained from independent blood samples of the same donor. EVs were prepared by ultracentrifugation. Dashed coloured boxes in the stain-free image correspond to the respective areas into which the blot was cut for incubation with different antibodies.

## Supplementary Figure S5


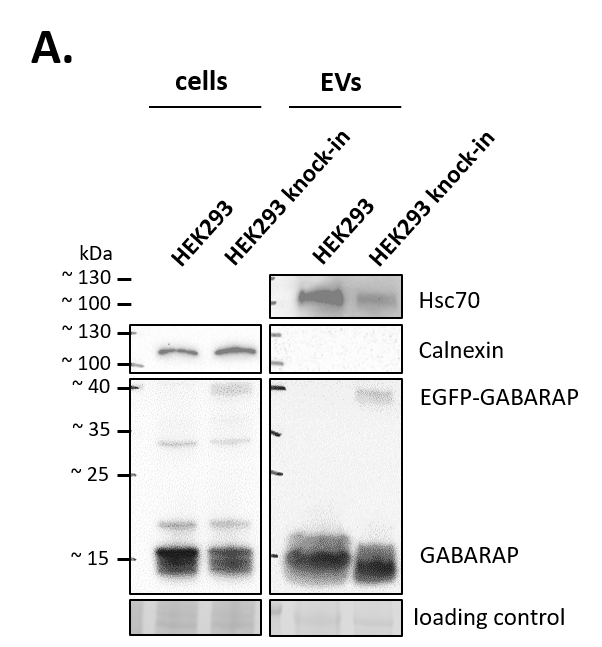


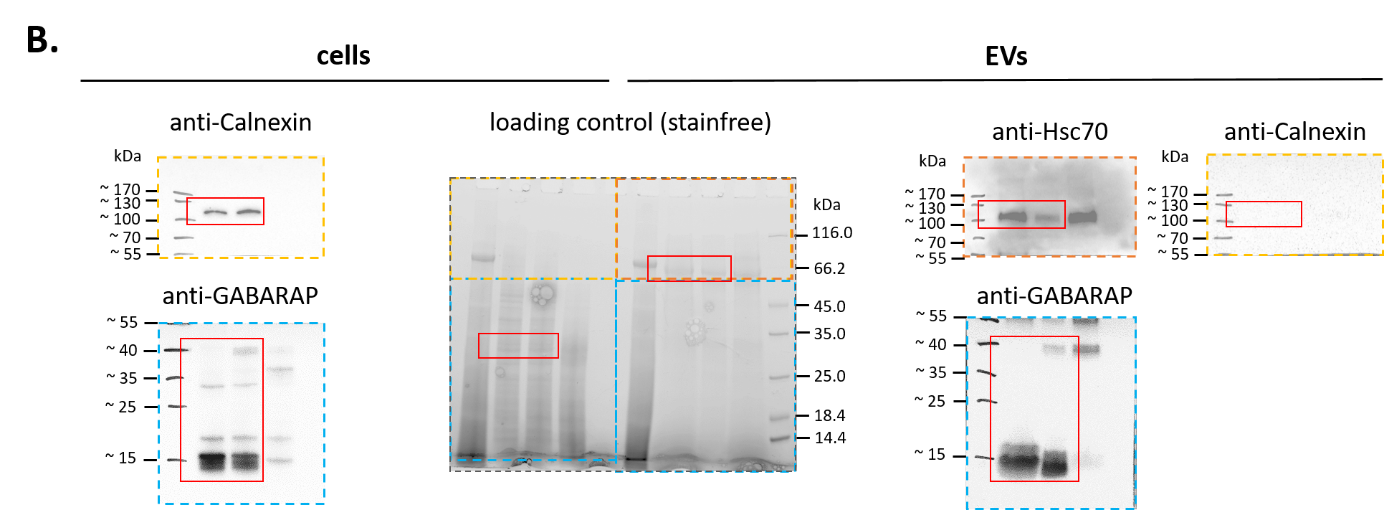


Supplementary Figure S5. EGFP-GABARAP, expressed on endogenous level [46], is secreted in EVs. (A) Wild-type HEK293 cells and knock-in HEK293 cells expressing EGFP-GABARAP on endogenous levels and their supernatants were harvested after an incubation period of 72 h. EVs were prepared by ultracentrifugation. In cellular and EV samples, both wild-type GABARAP and knocked-in EGFP-GABARAP were detected. A representative crop of a stain-free gel is shown as loading control. One representative blot out of two, each performed with lysates from different passage numbers, is shown. (B) Uncropped versions of the blots and the corresponding stain-free gel given in (A). Dashed coloured boxes in the stain-free gel correspond to the respective areas into which the blot was cut for incubation with different antibodies.

## Supplementary Figure S6
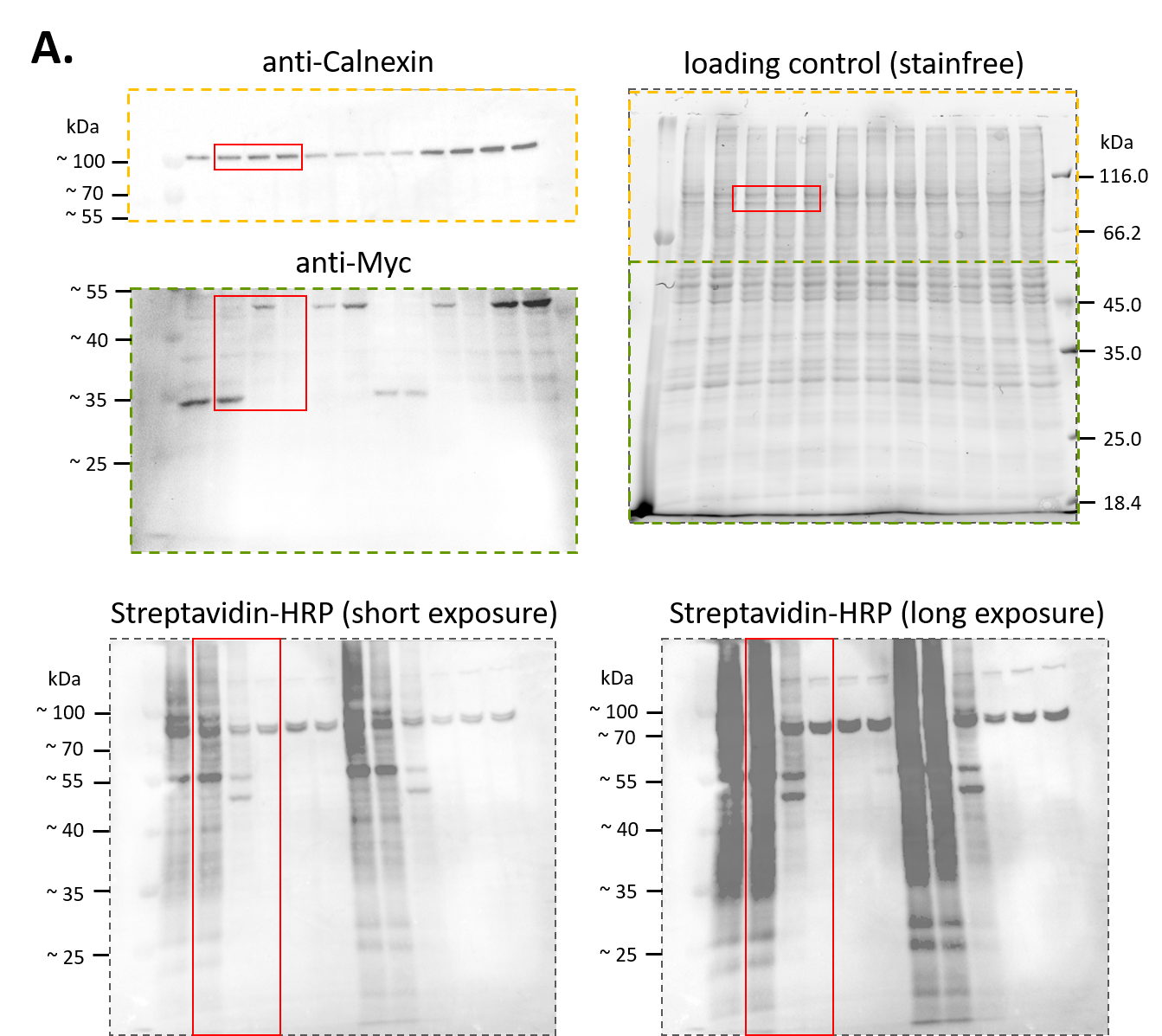


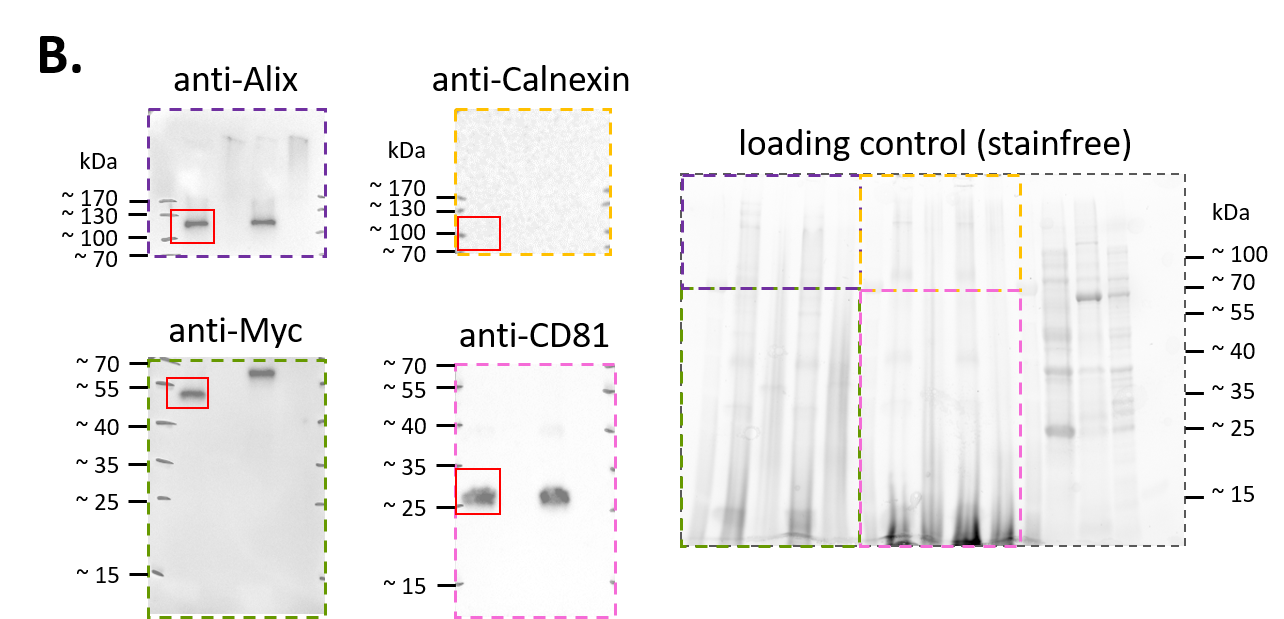


**Supplementary Figure S6.** Full images of blots shown in Figure 2B and 2D. The cropped region is indicated by red box. Cellular distribution of APEX2 and APEX2-GABARAP. (A) Uncropped versions of the blots and the stain-free gel related to Figure 2B. Different biotinylation patterns are obtained when expressing APEX2-GABARAP compared to APEX2. HEK293 cells were transfected with APEX2 and APEX2-GABARAP encoding plasmids, resp. For the negative control (neg ctrl), no DNA was added. After an incubation period of 48 h, cells were labelled, harvested, and lysed as described by Hung et al. [38]. Cellular lysates were subsequently analysed by immunoblotting using a Streptavidin-HRP conjugate. One representative blot of four different cellular passages is shown. (B) Uncropped versions of the blots and the stain-free gel related to Figure 2D. EVs were prepared from cell culture supernatant using polymer-based precipitation after 48 h cultivation and applied to immunoblotting. Both the absence of Calnexin (dashed yellow box) as cellular marker protein and the presence of common EV marker proteins (Alix (dashed purple box), CD81 (dashed magenta box)) and of APEX2-GABARAP itself (detected by anti-Myc antibody, dashed light green box) were confirmed. One representative blot of three different cellular passages is shown. Dashed coloured boxes in the stain-free image correspond to the respective areas into which the blot was cut for incubation with different antibodies.

## Supplementary Figure S7

##
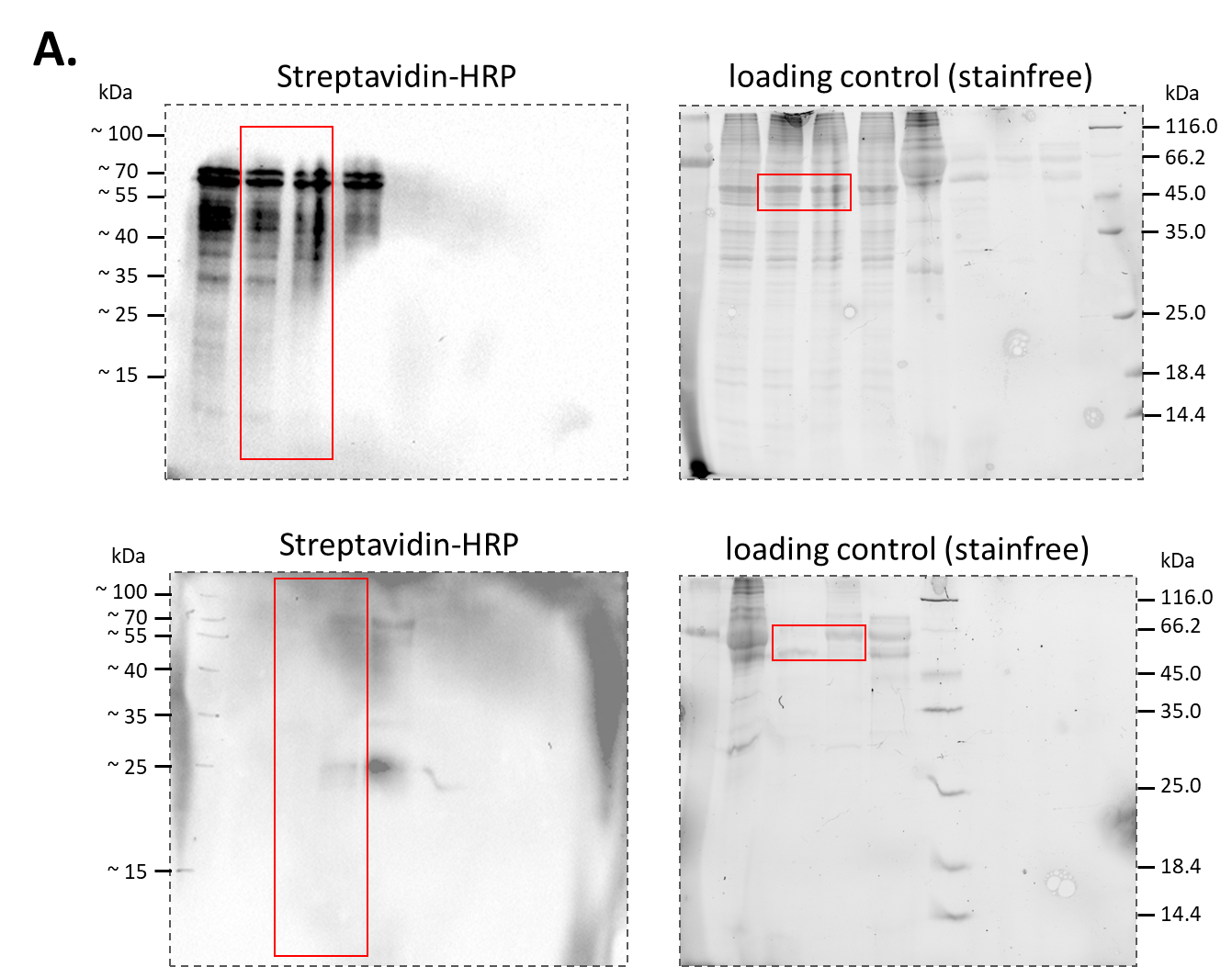


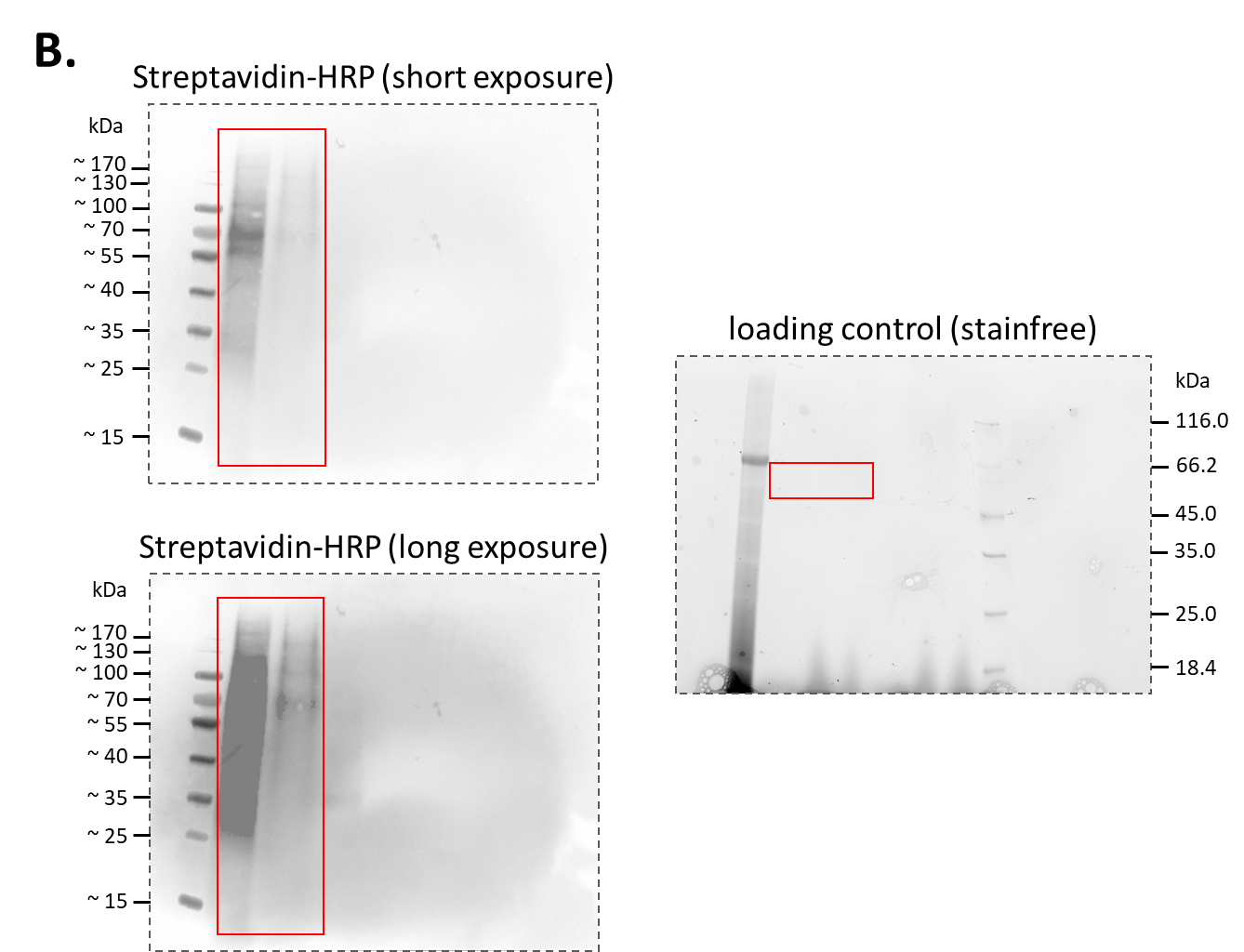


**Supplementary Figure S7.** Full images of blots shown in Figure 3B-3C. The cropped region is indicated by red box. APEX2-mediated proximity labelling in EVs. (A) Uncropped versions of the blots and the corresponding stain-free gels related to Figure 3B. Pelleted EVs, but not EVs in solution may be labelled by APEX2-GABARAP. The labelled EVs were lysed and used for immunoblotting, proving approach “b“ suitable for APEX2-mediated labelling in EVs. One representative blot of three different cellular passages is shown. (B) Uncropped versions of the blots and the corresponding stain-free gel related to Figure 3C. Biotinylated APEX2-GABARAP EV proteins, obtained using branch b, are enriched by Streptavidin. EV lysates were incubated with Streptavidin-coated beads. After collecting the flow-through (FT), multiple washing steps were applied, and the eluate (E) was collected. A broad signal was obtained for the eluate, while for the flow-through only faint signals were obtained, demonstrating effective capturing of biotinylated EV proteins. One representative blot of two different cellular passages is shown.

## Supplementary Figure S8

##
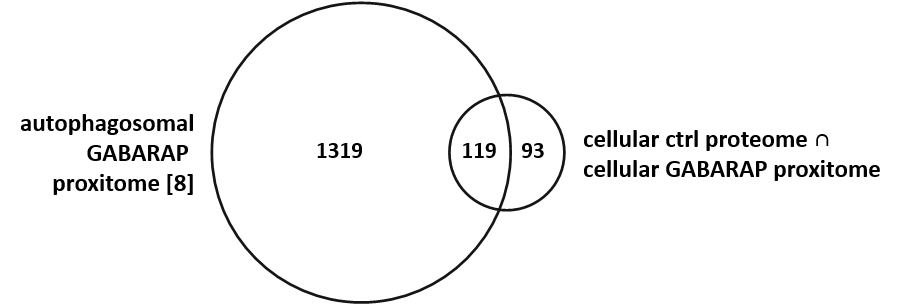


Supplementary Figure S8. Venn diagram of autophagosomal and cellular proteins. Comparison of the autophagosomal GABARAP proxitome as determined by Le Guerroué et al. [8] with the intersection between the unlabelled cellular ctrl proteome and the cellular GABARAP proxitome as defined in Figure 4A, consisting of 212 proteins (61.1 % of the cellular GABARAP proxitome) probably representing true-positive hits. Venn diagrams were created using FunRich [47, 48].

## Supplementary Figure S9


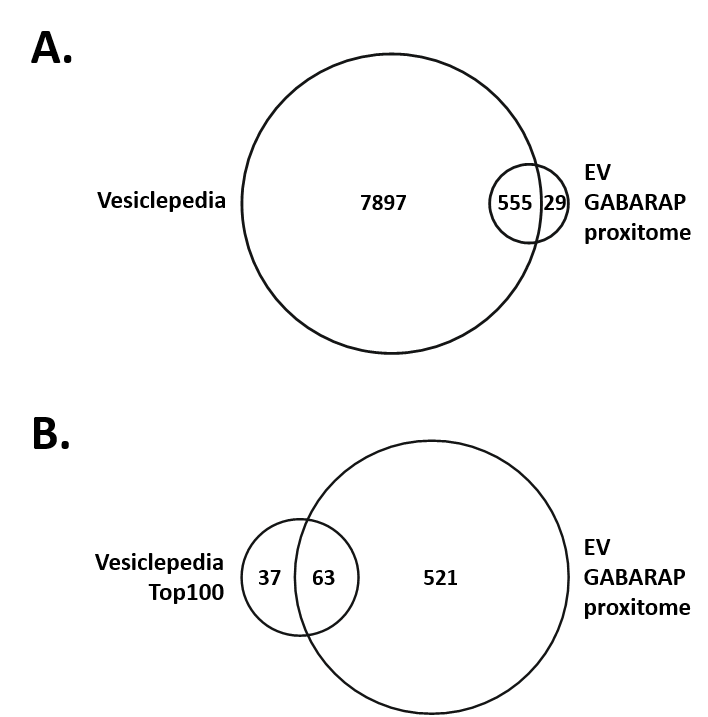


**Supplementary Figure S9.** Comparison of the EV GABARAP proxitome with Vesiclepedia entries [28, 29]. Of the EV GABARAP proxitome, more than 95 % are registered in Vesiclepedia (A) and 63 proteins were found in the Vesiclepedia top 100 (B). Either the complete database (A) or the Top100 entries (B) were used as assessed on Nov 12^th^, 2019. Venn diagrams were created using FunRich [47, 48].

## Supplementary Figure S10


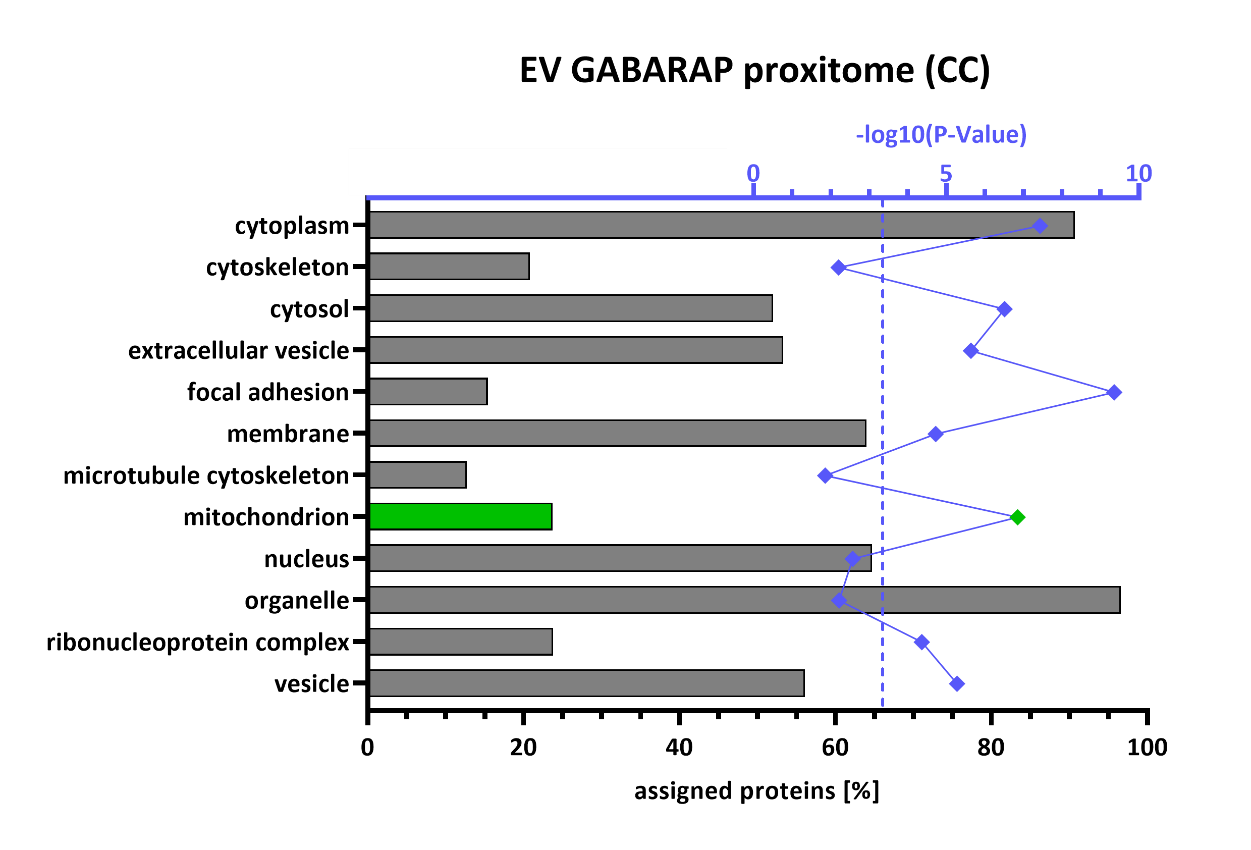


Supplementary Figure S10. Categorisation of the EV GABARAP proxitome using proteins of Table S1A as background. The obtained proteins for the EV GABARAP proxitome were analysed by GO cellular component (CC) using DAVID Bioinformatics Resources 6.8 [41]. Supplementary Table S1A, containing the complete list of proteins detected in this study, was set as background. The ratio of assigned proteins to the total protein number is depicted in [%]. In blue, the negative log10 of the P-Value is shown. Significantly overrepresented cellular compartments are defined by a P-Value of ≤ 0.05 % (dashed line). The mitochondrion-related bar [%] and -log10(P-Value) data point are highlighted in green.

## Supplementary Figure S11


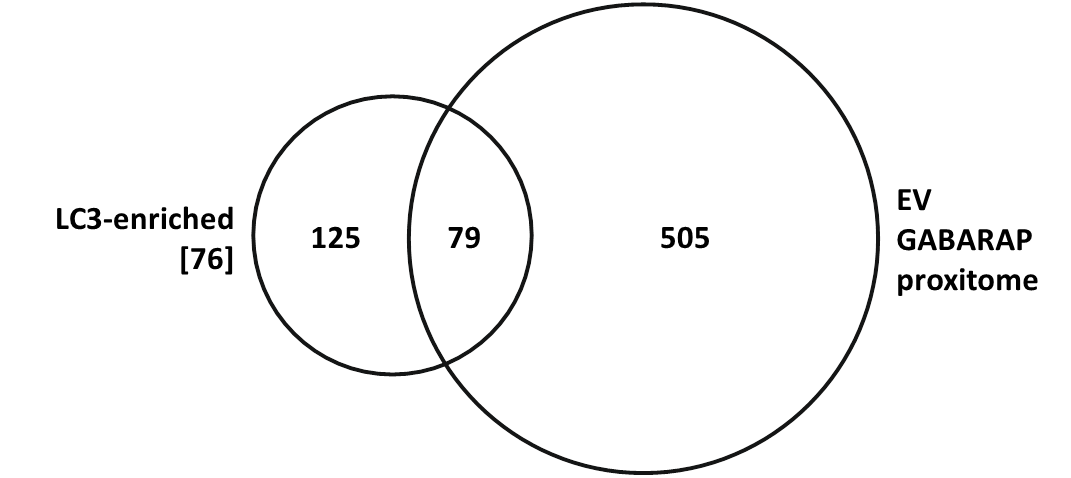


Supplementary Figure S11. Comparison with BirA*-LC3 enriched proteins. Venn diagram of the EV GABARAP proxitome with proteins significantly enriched with extravesicular BirA*-LC3 as defined by Leidal *et al.* [76]. Of the BirA*-LC3-enriched proteins, 38.7 % were also identified in the EV GABARAP proxitome. Venn diagrams were created using FunRich [47, 48].
