# Supplementary material for: The GABARAP Co-Secretome Identified by APEX2-GABARAP Proximity Labelling of Extracellular Vesicles
Source: Cells. 2020 Jun 16;9(6):1468. doi: 10.3390/cells9061468 (PMC7349886; doi:10.3390/cells9061468)
Supplement: Supplementary file 1 [file cells-09-01468-s001.zip › Supplementary data/APEX2-GABARAP_Supplemental_TableS2+S3.docx]

# Supplementary Information

**The GABARAP co-secretome identified by APEX2-GABARAP proximity labelling of extracellular vesicles**

Julia L. Sanwald ^1,2^, Gereon Poschmann ^3^, Kai Stühler ^3,4^, Christian Behrends ^5^, Silke Hoffmann ^2^, Dieter Willbold ^1,2^

^1^ Institut für Physikalische Biologie, Heinrich-Heine-Universität Düsseldorf, Universitätsstraße 1, 40225 Düsseldorf, Germany

^2^ Institute of Biological Information Processing (IBI-7: Structural Biochemistry), Forschungszentrum Jülich, Leo-Brandt-Straße, 52428 Jülich, Germany

^3^ Institute of Molecular Medicine I, Heinrich-Heine-Universität Düsseldorf, Universitätsstraße 1, 40225 Düsseldorf, Germany

^4^ Molecular Proteomics Laboratory, Biologisch-Medizinisches Forschungszentrum (BMFZ), Heinrich-Heine-Universität Düsseldorf, Universitätsstraße 1, 40225 Düsseldorf, Germany

^5^ Munich Cluster for Systems Neurology (SyNergy), Ludwig‐Maximilians‐Universität München, Feodor-Lynen-Straße 17, 81377 München, Germany

Supplementary Table S2 is related to Figure 4B, 4D, and 4E, and Figure 5A, 5B, 5E, 5F, and 5G

Supplementary Table S3 is related to Figure 4D

**Supplementary Table S2**

Supplementary Table S2. Proteins enriched or exclusive for the EV GABARAP proxitome. List and characterisation of proteins either enriched > 2fold or exclusively detected in the EV GABARAP proxitome compared to the EV ctrl proteome and the 293T exosome proteome [52] (intensity threshold > 24).

|  | **Uniprot accession** | **Entry name** | **Protein name** | **x-fold enrich-ment** | **Vesicle-pedia entry** | **Vesicle-pedia top 100 entry** | **xLIR** | **IntAct** | **Autophago-some GABARAP proxitome** | **RNA binding** | **Mito-chon-drial** |
| --- | --- | --- | --- | --- | --- | --- | --- | --- | --- | --- | --- |
| **> 2-fold enriched** | P04075 | ALDOA_HUMAN | Fructose-bisphosphate aldolase A | 5,520 | yes | yes |  |  | yes | yes |  |
|  | *APEX2-GABARAP* | | | 13,774 |  |  |  |  |  |  |  |
|  | P05023 | AT1A1_HUMAN | Sodium/potassium-transporting ATPase subunit alpha-1 | 39,233 | yes | yes | yes |  | yes |  |  |
|  | P11021 | BIP_HUMAN | Endoplasmic reticulum chaperone BiP | 2,777 | yes | yes | yes | yes | yes |  |  |
|  | P10809 | CH60_HUMAN | 60 kDa heat shock protein, mitochondrial | 131,219 | yes |  | yes | yes | yes | yes | yes |
|  | P23528 | COF1_HUMAN | Cofilin-1 | 7,767 | yes | yes |  |  | yes |  |  |
|  | Q9NR30 | DDX21_HUMAN | Nucleolar RNA helicase 2 | 4,855 | yes |  | yes | yes | yes | yes | yes |
|  | O00571 | DDX3X_HUMAN | ATP-dependent RNA helicase DDX3X | 4,140 | yes |  | yes | yes | yes | yes | yes |
|  | O15523 | DDX3Y_HUMAN | ATP-dependent RNA helicase DDX3Y | 4,140 | yes |  |  |  | yes | yes |  |
|  | P17844 | DDX5_HUMAN | Probable ATP-dependent RNA helicase DDX5 | 2,609 | yes |  |  | yes |  | yes |  |
|  | Q13838 | DX39B_HUMAN | Spliceosome RNA helicase DDX39B | 3,367 | yes |  | yes |  | yes | yes |  |
|  | P06733 | ENOA_HUMAN | Alpha-enolase | 10,697 | yes | yes |  | yes | yes | yes |  |
|  | P14625 | ENPL_HUMAN | Endoplasmin | 41,126 | yes |  | yes | yes | yes | yes |  |
|  | P15311 | EZRI_HUMAN | Ezrin | 3,386 | yes | yes | yes |  | yes | yes |  |
|  | Q13642 | FHL1_HUMAN | Four and a half LIM domains protein 1 | 2,156 | yes |  |  |  |  |  |  |
|  | Q16658 | FSCN1_HUMAN | Fascin | 2,229 | yes |  |  |  | yes | yes |  |
|  | P04406 | G3P_HUMAN | Glyceraldehyde-3-phosphate dehydrogenase | 2,004 | yes | yes |  | yes | yes |  |  |
|  | Q14697 | GANAB_HUMAN | Neutral alpha-glucosidase AB | 8,497 | yes |  | yes |  | yes | yes |  |
|  | P50395 | GDIB_HUMAN | Rab GDP dissociation inhibitor beta | 2,474 | yes | yes | yes |  | yes | yes |  |
|  | P61978 | HNRPK_HUMAN | Heterogeneous nuclear ribonucleoprotein K | 5,023 | yes |  |  | yes | yes | yes |  |
| **> 2-fold enriched** | P52272 | HNRPM_HUMAN | Heterogeneous nuclear ribonucleoprotein M | 8,855 | yes |  |  | yes | yes | yes |  |
|  | Q00839 | HNRPU_HUMAN | Heterogeneous nuclear ribonucleoprotein U | 21,111 | yes |  | yes | yes | yes | yes |  |
|  | O95757 | HS74L_HUMAN | Heat shock 70 kDa protein 4L | 2,512 | yes |  |  |  |  |  |  |
|  | P34932 | HSP74_HUMAN | Heat shock 70 kDa protein 4 | 4,756 | yes |  |  | yes | yes |  |  |
|  | P17066 | HSP76_HUMAN | Heat shock 70 kDa protein 6 | 3,939 | yes |  |  | yes |  |  |  |
|  | P11142 | HSP7C_HUMAN | Heat shock cognate 71 kDa protein | 3,066 | yes | yes |  | yes | yes | yes |  |
|  | Q12905 | ILF2_HUMAN | Interleukin enhancer-binding factor 2 | 3,262 | yes |  |  | yes |  | yes |  |
|  | Q12906 | ILF3_HUMAN | Interleukin enhancer-binding factor 3 | 3,590 | yes |  |  | yes | yes | yes |  |
|  | O00410 | IPO5_HUMAN | Importin-5 | 2,186 | yes |  | yes | yes | yes | yes |  |
|  | P33176 | KINH_HUMAN | Kinesin-1 heavy chain | 2,620 | yes |  | yes |  | yes |  |  |
|  | P00338 | LDHA_HUMAN | L-lactate dehydrogenase A chain | 6,198 | yes | yes | yes |  | yes |  |  |
|  | P07195 | LDHB_HUMAN | L-lactate dehydrogenase B chain | 3,598 | yes | yes | yes |  | yes |  |  |
|  | P25205 | MCM3_HUMAN | DNA replication licensing factor MCM3 | 2,899 | yes |  |  | yes | yes |  |  |
|  | P33991 | MCM4_HUMAN | DNA replication licensing factor MCM4 | 2,513 | yes |  |  |  |  |  |  |
|  | P30419 | NMT1_HUMAN | Glycylpeptide N-tetradecanoyltransferase 1 | 3,365 | yes |  |  |  |  |  |  |
|  | P19338 | NUCL_HUMAN | Nucleolin | 5,573 | yes |  | yes | yes | yes | yes |  |
|  | Q9UQ80 | PA2G4_HUMAN | Proliferation-associated protein 2G4 | 6,046 | yes |  |  |  | yes | yes |  |
|  | P11940 | PABP1_HUMAN | Polyadenylate-binding protein 1 | 2,039 | yes |  |  | yes | yes | yes |  |
|  | Q9H361 | PABP3_HUMAN | Polyadenylate-binding protein 3 | 2,039 | yes |  |  |  |  | yes |  |
|  | P09874 | PARP1_HUMAN | Poly [ADP-ribose] polymerase 1 | 2,649 | yes |  |  |  |  | yes |  |
|  | Q15365 | PCBP1_HUMAN | Poly(rC)-binding protein 1 | 2,743 | yes |  |  | yes | yes | yes |  |
|  | P30101 | PDIA3_HUMAN | Protein disulfide-isomerase A3 | 5,453 | yes |  |  |  | yes | yes |  |
|  | P00558 | PGK1_HUMAN | Phosphoglycerate kinase 1 | 3,239 | yes | yes | yes |  | yes |  |  |
|  | P13797 | PLST_HUMAN | Plastin-3 | 2,171 | yes |  | yes |  | yes |  |  |
|  | P62937 | PPIA_HUMAN | Peptidyl-prolyl cis-trans isomerase A | 33,083 | yes | yes |  | yes | yes | yes |  |
|  | Q06830 | PRDX1_HUMAN | Peroxiredoxin-1 | 2,856 | yes | yes | yes | yes | yes | yes |  |
|  | P60891 | PRPS1_HUMAN | Ribose-phosphate pyrophosphokinase 1 | 2,392 | yes |  |  |  | yes |  |  |
| **> 2-fold enriched** | P26599 | PTBP1_HUMAN | Polypyrimidine tract-binding protein 1 | 6,344 | yes |  |  | yes | yes | yes |  |
|  | O15067 | PUR4_HUMAN | Phosphoribosylformylglycinamidine synthase | 4,043 | yes |  |  |  |  |  |  |
|  | P30050 | RL12_HUMAN | 60S ribosomal protein L12 | 2,994 | yes |  |  | yes | yes | yes |  |
|  | Q07020 | RL18_HUMAN | 60S ribosomal protein L18 | 13,655 | yes |  |  | yes | yes | yes |  |
|  | P35268 | RL22_HUMAN | 60S ribosomal protein L22 | 7,201 | yes |  |  | yes | yes | yes |  |
|  | P46777 | RL5_HUMAN | 60S ribosomal protein L5 | 2,004 | yes |  |  | yes | yes | yes |  |
|  | Q02878 | RL6_HUMAN | 60S ribosomal protein L6 | 13,705 | yes |  |  | yes | yes | yes |  |
|  | P22626 | ROA2_HUMAN | Heterogeneous nuclear ribonucleoproteins A2/B1 | 4,378 | yes |  |  | yes | yes | yes |  |
|  | P62249 | RS16_HUMAN | 40S ribosomal protein S16 | 4,407 | yes |  |  | yes | yes | yes |  |
|  | P62701 | RS4X_HUMAN | 40S ribosomal protein S4, X isoform | 2,901 | yes |  | yes | yes | yes | yes |  |
|  | P62241 | RS8_HUMAN | 40S ribosomal protein S8 | 23,108 | yes |  |  | yes | yes | yes |  |
|  | O43175 | SERA_HUMAN | D-3-phosphoglycerate dehydrogenase | 2,181 | yes |  |  |  | yes |  |  |
|  | Q9Y5B9 | SP16H_HUMAN | FACT complex subunit SPT16 | 4,003 | yes |  | yes |  | yes | yes |  |
|  | P31948 | STIP1_HUMAN | Stress-induced-phosphoprotein 1 | 2,294 | yes |  |  |  | yes | yes |  |
|  | Q9P2J5 | SYLC_HUMAN | Leucine--tRNA ligase, cytoplasmic | 2,351 | yes |  |  |  | yes |  |  |
|  | P68363 | TBA1B_HUMAN | Tubulin alpha-1B chain | 9,567 | yes |  |  |  |  | yes |  |
|  | P68366 | TBA4A_HUMAN | Tubulin alpha-4A chain | 9,567 | yes |  |  | yes | yes |  |  |
|  | P68371 | TBB4B_HUMAN | Tubulin beta-4B chain | 9,098 | yes | yes |  | yes | yes | yes |  |
|  | Q13263 | TIF1B_HUMAN | Transcription intermediary factor 1-beta | 7,729 | yes |  |  |  | yes | yes |  |
|  | P29401 | TKT_HUMAN | Transketolase | 7,472 | yes |  |  |  | yes |  |  |
|  | P22314 | UBA1_HUMAN | Ubiquitin-like modifier-activating enzyme 1 | 3,362 | yes | yes | yes |  | yes | yes | yes |
| **Exclusi-vely EV GABA-RAP proxi-tome** | O95831 | AIFM1_HUMAN | Apoptosis-inducing factor 1, mitochondrial | - | yes |  |  | yes | yes |  | yes |
|  | P52895 | AK1C2_HUMAN | Aldo-keto reductase family 1 member C2 | - | yes |  |  |  |  |  |  |
|  | P42330 | AK1C3_HUMAN | Aldo-keto reductase family 1 member C3 | - | yes |  |  |  |  |  |  |
| **Exclusi-vely EV GABA-RAP proxi-tome** | P16615 | AT2A2_HUMAN | Sarcoplasmic/endoplasmic reticulum calcium ATPase 2 | - | yes |  | yes |  | yes |  |  |
|  | P48047 | ATPO_HUMAN | ATP synthase subunit O, mitochondrial | - | yes |  |  |  | yes |  | yes |
|  | Q6UB35 | C1TM_HUMAN | Monofunctional C1-tetrahydrofolate synthase, mitochondrial | - | yes |  |  |  | yes |  | yes |
|  | P00918 | CAH2_HUMAN | Carbonic anhydrase 2 | - | yes |  |  |  |  |  |  |
|  | P27797 | CALR_HUMAN | Calreticulin | - | yes |  | yes | yes | yes | yes |  |
|  | Q9UJS0 | CMC2_HUMAN | Solute carrier family 25 member 13 | - | yes |  | yes |  | yes |  | yes |
|  | Q9UBM7 | DHCR7_HUMAN | 7-dehydrocholesterol reductase | - | yes |  |  |  | yes |  |  |
|  | Q9BTC0 | DIDO1_HUMAN | Death-inducer obliterator 1 | - | yes |  |  |  |  | yes |  |
|  | P09622 | DLDH_HUMAN | Dihydrolipoyl dehydrogenase, mitochondrial | - | yes |  |  |  | yes |  | yes |
|  | P33316 | DUT_HUMAN | Deoxyuridine 5'-triphosphate nucleotidohydrolase, mitochondrial | - | yes |  |  | yes | yes | yes | yes |
|  | Q13011 | ECH1_HUMAN | Delta(3,5)-Delta(2,4)-dienoyl-CoA isomerase, mitochondrial | - | yes |  |  |  | yes |  | yes |
|  | P07954 | FUMH_HUMAN | Fumarate hydratase, mitochondrial | - | yes |  |  |  | yes |  | yes |
|  | Q99714 | HCD2_HUMAN | 3-hydroxyacyl-CoA dehydrogenase type-2 | - | yes |  |  | yes | yes | yes | yes |
|  | P23588 | IF4B_HUMAN | Eukaryotic translation initiation factor 4B | - | yes |  |  |  |  | yes |  |
|  | P05783 | K1C18_HUMAN | Keratin, type I cytoskeletal 18 | - | yes |  |  |  | yes | yes |  |
|  | Q14739 | LBR_HUMAN | Delta(14)-sterol reductase LBR | - | yes |  |  |  |  | yes |  |
|  | Q9UGP4 | LIMD1_HUMAN | LIM domain-containing protein 1 | - |  |  |  |  |  |  |  |
|  | Q86UE4 | LYRIC_HUMAN | Protein LYRIC | - | yes |  |  | yes |  | yes |  |
|  | P27816 | MAP4_HUMAN | Microtubule-associated protein 4 | - | yes |  |  |  |  | yes |  |
|  | Q16891 | MIC60_HUMAN | MICOS complex subunit MIC60 | - | yes |  |  |  | yes | yes | yes |
|  | Q10713 | MPPA_HUMAN | Mitochondrial-processing peptidase subunit alpha | - | yes |  | yes |  | yes |  | yes |
|  | Q13423 | NNTM_HUMAN | NAD(P) transhydrogenase, mitochondrial | - | yes |  |  |  | yes |  | yes |
|  | P15559 | NQO1_HUMAN | NAD(P)H dehydrogenase [quinone] 1 | - | yes |  | yes |  | yes | yes |  |
|  | P04181 | OAT_HUMAN | Ornithine aminotransferase, mitochondrial | - | yes |  | yes |  | yes |  | yes |
| **Exclusi-vely EV GABA-RAP proxi-tome** | P08559 | ODPA_HUMAN | Pyruvate dehydrogenase E1 component subunit alpha, somatic form, mitochondrial | - | yes |  |  |  | yes |  | yes |
|  | Q04941 | PLP2_HUMAN | Proteolipid protein 2 | - | yes |  |  |  |  |  |  |
|  | P48634 | PRC2A_HUMAN | Protein PRRC2A | - | yes |  |  |  |  |  |  |
|  | P31930 | QCR1_HUMAN | Cytochrome b-c1 complex subunit 1, mitochondrial | - | yes |  | yes |  | yes |  | yes |
|  | Q96PK6 | RBM14_HUMAN | RNA-binding protein 14 | - | yes |  |  | yes |  | yes |  |
|  | O76021 | RL1D1_HUMAN | Ribosomal L1 domain-containing protein 1 | - | yes |  |  | yes | yes | yes |  |
|  | P83881 | RL36A_HUMAN | 60S ribosomal protein L36a | - | yes |  |  |  | yes | yes |  |
|  | P31040 | SDHA_HUMAN | Succinate dehydrogenase [ubiquinone] flavoprotein subunit, mitochondrial | - | yes |  | yes | yes | yes |  | yes |
|  | P24752 | THIL_HUMAN | Acetyl-CoA acetyltransferase, mitochondrial | - | yes |  |  |  | yes |  | yes |

**Supplementary Table S3.**

**Supplementary Table S3.** Proteins containing an xLIR motif. Output of the xLIR motif search in proteins found in the intersection EV GABARAP proxitome ∩ autophagosome GABARAP proxitome (376 proteins). In 164 proteins, 271 hits were detected. For the query, the xLIR motif [ADEFGLPRSK]-[DEGMSTV]-[WFY]-[DEILQTV]-[ADEFHIKLMPSTV]-[ILV] was used. The online tool ScanProsite was applied to enable a search in protein lists.

| **Uniprot accession** | **Entry name** | **Protein name** | **Position** | | **Sequence** |
| --- | --- | --- | --- | --- | --- |
| Q13085-3 | ACACA_HUMAN | Acetyl-CoA carboxylase 1 | 1554 | 1559 | LTYTEL |
|  |  |  | 1813 | 1818 | GVFTVL |
|  |  |  | 1947 | 1952 | KTYQAI |
|  |  |  | 2013 | 2018 | GSWVVI |
|  |  |  | 2064 | 2069 | PVYIHL |
| P53396-2 | ACLY_HUMAN | ATP-citrate synthase | 197 | 202 | FTYLEI |
|  |  |  | 778 | 783 | RSFDEL |
|  |  |  | 789 | 794 | SVYEDL |
|  |  |  | 1039 | 1044 | DEYIDI |
| P62736 | ACTA_HUMAN | Actin, aortic smooth muscle | 133 | 138 | AMYVAI |
| P60709 | ACTB_HUMAN | Actin, cytoplasmic 1 | 131 | 136 | AMYVAI |
| Q562R1 | ACTBL_HUMAN | Beta-actin-like protein 2 | 132 | 137 | AMYVAI |
| P12814-2 | ACTN1_HUMAN | Alpha-actinin-1 | 277 | 282 | EDYEKL |
| O43707 | ACTN4_HUMAN | Alpha-actinin-4 | 296 | 301 | EDYEKL |
| P61158 | ARP3_HUMAN | Actin-related protein 3 | 398 | 403 | KDYEEI |
| O95433 | AHSA1_HUMAN | Activator of 90 kDa heat shock protein ATPase homolog 1 | 259 | 264 | GEFTDL |
| P48444-2 | COPD_HUMAN | Coatomer subunit delta | 283 | 288 | ESFIPL |
| P05023-3 | AT1A1_HUMAN | Sodium/potassium-transporting ATPase subunit alpha-1 | 385 | 390 | ATWLAL |
|  |  |  | 968 | 973 | FVYDEV |
| P16615-2 | AT2A2_HUMAN | Sarcoplasmic/endoplasmic reticulum calcium ATPase 2 | 432 | 437 | GVYEKV |
|  |  |  | 655 | 660 | REFDEL |
| P06576 | ATPB_HUMAN | ATP synthase subunit beta, mitochondrial | 497 | 502 | GEYDHL |
| Q9HB71-3 | CYBP_HUMAN | Calcyclin-binding protein | 68 | 73 | RSFDLL |
| P27797 | CALR_HUMAN | Calreticulin | 107 | 112 | GGYVKL |
|  |  |  | 166 | 171 | DEFTHL |
|  |  |  | 198 | 203 | DDWDFL |
| Q86VP6 | CAND1_HUMAN | Cullin-associated NEDD8-dissociated protein 1 | 1122 | 1127 | LTFLML |
| P27824 | CALX_HUMAN | Calnexin | 494 | 499 | PVFLVI |
| P52907 | CAZA1_HUMAN | F-actin-capping protein subunit alpha-1 | 226 | 231 | KEFIKI |
| P16152 | CBR1_HUMAN | Carbonyl reductase [NADPH] 1 | 251 | 256 | PVYLAL |
| P40227 | TCPZ_HUMAN | T-complex protein 1 subunit zeta | 287 | 292 | KGFVVI |
| Q99832-3 | TCPH_HUMAN | T-complex protein 1 subunit eta | 217 | 222 | EDYQAI |
| P06493 | CDK1_HUMAN | Cyclin-dependent kinase 1 | 2 | 7 | EDYTKI |
| Q07065 | CKAP4_HUMAN | Cytoskeleton-tassociated protein 4 | 170 | 175 | GTFESI |
|  |  |  | 228 | 233 | RDFTSL |
| Q00610-2 | CLH1_HUMAN | Clathrin heavy chain 1 | 512 | 517 | PDWIFL |
|  |  |  | 1315 | 1320 | GMFTEL |
|  |  |  | 1475 | 1480 | EDYQAL |
| P55060-4 | XPO2_HUMAN | Exportin-2 | 590 | 595 | LVFTEI |
|  |  |  | 676 | 681 | GVFQKL |
| P00387-2 | NB5R3_HUMAN | NADH-cytochrome b5 reductase 3 | 80 | 85 | KGFVDL |
| P39656-3 | OST48_HUMAN | Dolichyl-diphosphooligosaccharide-protein glycosyltransferase 48 kDa subunit | 379 | 384 | LGYTHL |
| Q9NR30 | DDX21_HUMAN | Nucleolar RNA helicase 2 | 545 | 550 | EEYQLV |
| Q13838 | DX39B_HUMAN | Spliceosome RNA helicase DDX39B | 37 | 42 | GSYVSI |
| O00571-2 | DDX3X_HUMAN | ATP-dependent RNA helicase DDX3X | 382 | 387 | DEYIFL |
| Q7L2E3-3 | DHX30_HUMAN | ATP-dependent RNA helicase DHX30 | 637 | 642 | PGWQEI |
|  |  |  | 905 | 910 | AGWEEV |
| Q08211 | DHX9_HUMAN | ATP-dependent RNA helicase A | 697 | 702 | KVFDPV |
|  |  |  | 1085 | 1090 | DDWIKL |
| P10515 | ODP2_HUMAN | Dihydrolipoyllysine-residue acetyltransferase component of pyruvate dehydrogenase complex, mitochondrial | 418 | 423 | GVFTDI |
| P15924-2 | DESP_HUMAN | Desmoplakin | 1153 | 1158 | LGWQKL |
| Q14204 | DYHC1_HUMAN | Cytoplasmic dynein 1 heavy chain 1 | 407 | 412 | EEFEKV |
|  |  |  | 425 | 430 | DEYEKL |
|  |  |  | 1381 | 1386 | ASYEFV |
|  |  |  | 1488 | 1493 | RGWDDL |
|  |  |  | 1535 | 1540 | DVWIDV |
|  |  |  | 2879 | 2884 | KDYIPV |
|  |  |  | 3639 | 3644 | ESYDPV |
| P13639 | EF2_HUMAN | Elongation factor 2 | 299 | 304 | KVFDAI |
| Q15029-2 | U5S1_HUMAN | 116 kDa U5 small nuclear ribonucleoprotein component | 810 | 815 | AVYTVL |
| P05198 | IF2A_HUMAN | Eukaryotic translation initiation factor 2 subunit 1 | 80 | 85 | KGYIDL |
| Q14152-2 | EIF3A_HUMAN | Eukaryotic translation initiation factor 3 subunit A | 7 | 12 | RTWQKI |
|  |  |  | 1341 | 1346 | DGWTTV |
| P55884 | EIF3B_HUMAN | Eukaryotic translation initiation factor 3 subunit B | 230 | 235 | KGYIFL |
| Q99613-2 | EIF3C_HUMAN | Eukaryotic translation initiation factor 3 subunit C | 81 | 86 | EEFELL |
|  |  |  | 443 | 448 | EEFTKI |
|  |  |  | 787 | 792 | SVYDSI |
| Q9Y262-2 | EIF3L_HUMAN | Eukaryotic translation initiation factor 3 subunit L | 126 | 131 | AVFLIL |
|  |  |  | 413 | 418 | RSFLKL |
|  |  |  | 428 | 433 | AGFLDL |
| P60842 | IF4A1_HUMAN | Eukaryotic initiation factor 4A-I | 68 | 73 | KGYDVI |
|  |  |  | 161 | 166 | RVFDML |
| Q14240 | IF4A2_HUMAN | Eukaryotic initiation factor 4A-II | 69 | 74 | KGYDVI |
|  |  |  | 162 | 167 | RVFDML |
| Q9GZV4 | IF5A2_HUMAN | Eukaryotic translation initiation factor 5A-2 | 96 | 101 | DGYLSL |
| P07814 | SYEP_HUMAN | Bifunctional glutamate/proline-tRNA ligase | 207 | 212 | SGYLHI |
|  |  |  | 246 | 251 | EDFEKV |
|  |  |  | 1250 | 1255 | KMFEIV |
|  |  |  | 1431 | 1436 | EDFQKI |
| P30040 | ERP29_HUMAN | Endoplasmic reticulum resident protein 29 | 19 | 24 | LGFLLL |
|  |  |  | 159 | 164 | PVYDAL |
| P15311 | EZRI_HUMAN | Ezrin | 56 | 61 | PTWLKL |
|  |  |  | 189 | 194 | LEYLKI |
|  |  |  | 476 | 481 | PVYEPV |
|  |  |  | 581 | 586 | DEFEAL |
| Q9NSD9-2 | SYFB_HUMAN | Phenylalanine-tRNA ligase beta subunit | 38 | 43 | DSFIEL |
|  |  |  | 407 | 412 | PGFEII |
| P49327 | FAS_HUMAN | Fatty acid synthase | 544 | 549 | STFDDI |
|  |  |  | 657 | 662 | PVFEFV |
|  |  |  | 1339 | 1344 | GGFLLL |
|  |  |  | 1694 | 1699 | RVFTTV |
| Q02790 | FKBP4_HUMAN | Peptidyl-prolyl cis-trans isomerase FKBP4 | 374 | 379 | ADFQKV |
| P21333-2 | FLNA_HUMAN | Filamin-A | 2616 | 2621 | GEYTLV |
| O75369-6 | FLNB_HUMAN | Filamin-B | 113 | 118 | LVWTLI |
|  |  |  | 1816 | 1821 | ATFTIV |
| Q14697 | GANAB_HUMAN | Neutral alpha-glucosidase AB | 320 | 325 | ETWVDI |
|  |  |  | 363 | 368 | DVFLLL |
|  |  |  | 621 | 626 | AEWDHL |
| P50395-2 | GDIB_HUMAN | Rab GDP dissociation inhibitor beta | 3 | 8 | EEYDVI |
| P00367 | DHE3_HUMAN | Glutamate dehydrogenase 1, mitochondrial | 127 | 132 | GSWEVI |
| P40939 | ECHA_HUMAN | Trifunctional enzyme subunit alpha, mitochondrial | 447 | 452 | AVFEDL |
| P19367-4 | HXK1_HUMAN | Hexokinase-1 | 66 | 71 | GDFIAL |
|  |  |  | 514 | 519 | GDFLAL |
| Q99729-3 | ROAA_HUMAN | Heterogeneous nuclear ribonucleoprotein A/B | 195 | 200 | RGFVFI |
| Q00839-2 | HNRPU_HUMAN | Heterogeneous nuclear ribonucleoprotein U | 452 | 457 | EEYTFI |
| Q9BUJ2-4 | HNRL1_HUMAN | Heterogeneous nuclear ribonucleoprotein U-like protein 1 | 295 | 300 | PGFTFI |
| P51659 | DHB4_HUMAN | Peroxisomal multifunctional enzyme type 2 | 114 | 119 | EDWDII |
|  |  |  | 626 | 631 | FVFEEI |
| P08238 | HS90B_HUMAN | Heat shock protein HSP 90-beta | 506 | 511 | RGFEVV |
| P14625 | ENPL_HUMAN | Endoplasmin | 256 | 261 | SDYLEL |
|  |  |  | 406 | 411 | SDYIKL |
|  |  |  | 561 | 566 | KGYEVI |
|  |  |  | 613 | 618 | KEFEPL |
| P11021 | BIP_HUMAN | Endoplasmic reticulum chaperone BiP | 240 | 245 | GVFEVV |
| P38646 | GRP75_HUMAN | Stress-70 protein, mitochondrial | 452 | 457 | GVFTKL |
| P10809 | CH60_HUMAN | 60 kDa heat shock protein, mitochondrial | 126 | 131 | EGFEKI |
| Q92598-2 | HS105_HUMAN | Heat shock protein 105 kDa | 87 | 92 | LSYDLV |
|  |  |  | 670 | 675 | KMFEEL |
| Q7Z6Z7-2 | HUWE1_HUMAN | E3 ubiquitin-protein ligase HUWE1 | 3267 | 3272 | LSWLSV |
|  |  |  | 4188 | 4193 | KEYVHL |
|  |  |  | 4335 | 4340 | ESFEKL |
| Q9Y4L1-2 | HYOU1_HUMAN | Hypoxia up-regulated protein 1 | 471 | 476 | SVFETL |
| P41252 | SYIC_HUMAN | Isoleucine-tRNA ligase, cytoplasmic | 271 | 276 | SDYEIL |
|  |  |  | 473 | 478 | DDFEEV |
| P11717 | MPRI_HUMAN | Cation-independent mannose-6-phosphate receptor | 839 | 844 | GSFTEV |
|  |  |  | 1016 | 1021 | EGFITL |
| O00410-2 | IPO5_HUMAN | Importin-5 | 595 | 600 | DGWEFV |
|  |  |  | 937 | 942 | LSWLPL |
| P46940 | IQGA1_HUMAN | Ras GTPase-activating-like protein IQGAP1 | 256 | 261 | STYQDI |
|  |  |  | 285 | 290 | DVYEEL |
|  |  |  | 1018 | 1023 | EEYLLL |
| P05556 | ITB1_HUMAN | Integrin beta-1 | 250 | 255 | GGFDAI |
|  |  |  | 339 | 344 | EEFQPV |
| Q15046 | SYK_HUMAN | Lysine-tRNA ligase | 167 | 172 | EEFIHI |
|  |  |  | 261 | 266 | LGFLEI |
|  |  |  | 449 | 454 | GEFLEV |
| P33176 | KINH_HUMAN | Kinesin-1 heavy chain | 514 | 519 | KEYELL |
| Q14974 | IMB1_HUMAN | Importin subunit beta-1 | 501 | 506 | SSFELI |
|  |  |  | 750 | 755 | SDYDMV |
| Q6PKG0-3 | LARP1_HUMAN | La-related protein 1 | 217 | 222 | ATYVPV |
|  |  |  | 356 | 361 | DGFLPI |
| P00338 | LDHA_HUMAN | L-lactate dehydrogenase A chain | 286 | 291 | DVFLSV |
| P07195 | LDHB_HUMAN | L-lactate dehydrogenase B chain | 287 | 292 | EVFLSL |
| O95202 | LETM1_HUMAN | Mitochondrial proton/calcium exchanger protein | 213 | 218 | LVFVVV |
| P36776-3 | LONM_HUMAN | Lon protease homolog, mitochondrial | 306 | 311 | LEFIAV |
| P42704 | LPPRC_HUMAN | Leucine-rich PPR motif-containing protein, mitochondrial | 268 | 273 | DTYLAL |
| P40925-2 | MDHC_HUMAN | Malate dehydrogenase, cytoplasmic | 119 | 124 | GVYEAL |
| Q13724-2 | MOGS_HUMAN | Mannosyl-oligosaccharide glucosidase | 583 | 588 | LGYVSL |
| P26038 | MOES_HUMAN | Moesin | 56 | 61 | STWLKL |
|  |  |  | 189 | 194 | LEYLKI |
| P11586 | C1TC_HUMAN | C-1-tetrahydrofolate synthase, cytoplasmic | 810 | 815 | SSFQLL |
| Q9BQG0 | MBB1A_HUMAN | Myb-binding protein 1A | 996 | 1001 | PMFLSL |
| P19338 | NUCL_HUMAN | Nucleolin | 509 | 514 | ATFIKV |
| P22392-2 | NDKB_HUMAN | Nucleoside diphosphate kinase B | 6 | 11 | RTFIAI |
|  |  |  | 121 | 126 | RTFIAI |
| P46087-2 | NOP2_HUMAN | Probable 28S rRNA (cytosine(4447)-C(5))-methyltransferase | 641 | 646 | ASFQKL |
| P15559-2 | NQO1_HUMAN | NAD(P)H dehydrogenase [quinone] 1 | 33 | 38 | KGWEVV |
| P04181 | OAT_HUMAN | Ornithine aminotransferase, mitochondrial | 202 | 207 | PGFDII |
| P22234 | PUR6_HUMAN | Multifunctional protein ADE2 | 20 | 25 | EVYELL |
| Q8WUM4 | PDC6I_HUMAN | Programmed cell death 6-interacting protein | 2 | 7 | ATFISV |
| P13667 | PDIA4_HUMAN | Protein disulfide-isomerase A4 | 99 | 104 | PEYEKI |
|  |  |  | 533 | 538 | KTFDSI |
| P00558-2 | PGK1_HUMAN | Phosphoglycerate kinase 1 | 214 | 219 | FTFLKV |
| O00264 | PGRC1_HUMAN | Membrane-associated progesterone receptor component 1 | 137 | 142 | DEYDDL |
| P35232 | PHB_HUMAN | Prohibitin | 4 | 9 | KVFESI |
| Q99623-2 | PHB2_HUMAN | Prohibitin-2 | 208 | 213 | PGYIKL |
| Q15149-7 | PLEC_HUMAN | Plectin | 277 | 282 | SSFEEI |
|  |  |  | 1112 | 1117 | PTFDAL |
| O00469 | PLOD2_HUMAN | Procollagen-lysine,2-oxoglutarate 5-dioxygenase 2 | 442 | 447 | EDYVDI |
| P13797-3 | PLST_HUMAN | Plastin-3 | 239 | 244 | SGWQKI |
| Q10713 | MPPA_HUMAN | Mitochondrial-processing peptidase subunit alpha | 501 | 506 | PTYEHI |
| P30153 | 2AAA_HUMAN | Serine/threonine-protein phosphatase 2A 65 kDa regulatory subunit A alpha isoform | 74 | 79 | GTFTTL |
| Q06830 | PRDX1_HUMAN | Peroxiredoxin-1 | 46 | 51 | LDFTFV |
| P32119 | PRDX2_HUMAN | Peroxiredoxin-2 | 45 | 50 | LDFTFV |
| P30048-2 | PRDX3_HUMAN | Thioredoxin-dependent peroxide reductase, mitochondrial | 84 | 89 | LDFTFV |
| Q13162 | PRDX4_HUMAN | Peroxiredoxin-4 | 118 | 123 | LDFTFV |
| P30041 | PRDX6_HUMAN | Peroxiredoxin-6 | 41 | 46 | RDFTPV |
| P78527-2 | PRKDC_HUMAN | DNA-dependent protein kinase catalytic subunit | 173 | 178 | KVYELL |
|  |  |  | 383 | 388 | FMYVEL |
|  |  |  | 429 | 434 | EVYTPV |
|  |  |  | 463 | 468 | KVFLAL |
|  |  |  | 525 | 530 | KDYVDL |
|  |  |  | 649 | 654 | FSYELI |
|  |  |  | 760 | 765 | LSYTPL |
|  |  |  | 1099 | 1104 | FVFEAL |
|  |  |  | 2934 | 2939 | GEYDVL |
|  |  |  | 4044 | 4049 | RDYVAV |
| Q99873-3 | ANM1_HUMAN | Protein arginine N-methyltransferase 1 | 302 | 307 | EDYLTV |
| O94906-2 | PRP6_HUMAN | Pre-mRNA-processing factor 6 | 155 | 160 | EEWLSI |
| Q6P2Q9 | PRP8_HUMAN | Pre-mRNA-processing-splicing factor 8 | 487 | 492 | LDWVEV |
|  |  |  | 983 | 988 | KMYEKI |
|  |  |  | 1072 | 1077 | LSFQDI |
|  |  |  | 1974 | 1979 | EEWIKV |
| Q9Y617-2 | SERC_HUMAN | Phosphoserine aminotransferase | 129 | 134 | GSYTKI |
| P61026 | RAB10_HUMAN | Ras-related protein Rab-10 | 4 | 9 | KTYDLL |
| Q15286-2 | RAB35_HUMAN | Ras-related protein Rab-35 | 3 | 8 | RDYDHL |
| P54136-2 | SYRC_HUMAN | Arginine-tRNA ligase, cytoplasmic | 156 | 161 | AGYDVL |
|  |  |  | 185 | 190 | PDYLTV |
|  |  |  | 575 | 580 | KGFDIL |
| P62750 | RL23A_HUMAN | 60S ribosomal protein L23a | 142 | 147 | PDYDAL |
| P83731 | RL24_HUMAN | 60S ribosomal protein L24 | 27 | 32 | KVFQFL |
| P61353 | RL27_HUMAN | 60S ribosomal protein L27 | 69 | 74 | KSFVKV |
| P18124 | RL7_HUMAN | 60S ribosomal protein L7 | 120 | 125 | GTFVKL |
| P04843 | RPN1_HUMAN | Dolichyl-diphosphooligosaccharide-protein glycosyltransferase subunit 1 | 183 | 188 | ESYTKL |
| P46783 | RS10_HUMAN | 40S ribosomal protein S10 | 80 | 85 | RDYLHL |
| P25398 | RS12_HUMAN | 40S ribosomal protein S12 | 59 | 64 | PMYVKL |
| P62263 | RS14_HUMAN | 40S ribosomal protein S14 | 39 | 44 | DTFVHV |
| P15880 | RS2_HUMAN | 40S ribosomal protein S2 | 239 | 244 | ATFDAI |
| P63220 | RS21_HUMAN | 40S ribosomal protein S21 | 6 | 11 | GEFVDL |
| P62701 | RS4X_HUMAN | 40S ribosomal protein S4, X isoform | 203 | 208 | GSFDVV |
| P62081 | RS7_HUMAN | 40S ribosomal protein S7 | 70 | 75 | KSFQKI |
| P08865 | RSSA_HUMAN | 40S ribosomal protein SA | 53 | 58 | RTWEKL |
| P23921 | RIR1_HUMAN | Ribonucleoside-diphosphate reductase large subunit | 309 | 314 | FEFLDL |
|  |  |  | 365 | 370 | EEFEKL |
|  |  |  | 632 | 637 | GEFQIV |
| Q9Y265 | RUVB1_HUMAN | RuvB-like 1 | 218 | 223 | EEYVPL |
| P31040-2 | SDHA_HUMAN | Succinate dehydrogenase [ubiquinone] flavoprotein subunit, mitochondrial | 579 | 584 | LSYVDV |
| O75533 | SF3B1_HUMAN | Splicing factor 3B subunit 1 | 419 | 424 | AGYVPI |
|  |  |  | 722 | 727 | ESFDSV |
| Q15393 | SF3B3_HUMAN | Splicing factor 3B subunit 3 | 75 | 80 | KDYIVV |
|  |  |  | 734 | 739 | LSYETL |
|  |  |  | 1039 | 1044 | LDYDTV |
|  |  |  | 1117 | 1122 | LVYTTL |
| P23246 | SFPQ_HUMAN | Splicing factor, proline- and glutamine-rich | 334 | 339 | FGFIKL |
| P34897-3 | GLYM_HUMAN | Serine hydroxymethyltransferase, mitochondrial | 34 | 39 | EMWELL |
|  |  |  | 128 | 133 | AVYTAL |
| Q9UJS0 | CMC2_HUMAN | Calcium-binding mitochondrial carrier protein Aralar2 | 598 | 603 | LTYELL |
| O95347-2 | SMC2_HUMAN | Structural maintenance of chromosomes protein 2 | 218 | 223 | LEYQKV |
|  |  |  | 494 | 499 | ETYEAL |
| Q9NTJ3-2 | SMC4_HUMAN | Structural maintenance of chromosomes protein 4 | 173 | 178 | DDYEVI |
|  |  |  | 640 | 645 | LDYIVV |
| O75643 | U520_HUMAN | U5 small nuclear ribonucleoprotein 200 kDa helicase | 85 | 90 | KGYTLL |
|  |  |  | 111 | 116 | ETYEVL |
|  |  |  | 315 | 320 | FDFIKV |
|  |  |  | 440 | 445 | KGYEEV |
|  |  |  | 1257 | 1262 | PVFEPL |
|  |  |  | 1755 | 1760 | LTWTFL |
|  |  |  | 2019 | 2024 | LSYEVV |
| P05455 | LA_HUMAN | Lupus La protein | 44 | 49 | EGWVPL |
| Q9Y5B9 | SP16H_HUMAN | FACT complex subunit SPT16 | 157 | 162 | EGFDKI |
|  |  |  | 988 | 993 | KDWDEL |
| P26639 | SYTC_HUMAN | Threonine-tRNA ligase 1, cytoplasmic | 367 | 372 | RGFQEV |
| P02786 | TFR1_HUMAN | Transferrin receptor protein 1 | 241 | 246 | KDFEDL |
| Q9Y490 | TLN1_HUMAN | Talin-1 | 257 | 262 | AGFLDL |
|  |  |  | 393 | 398 | AGYIDI |
|  |  |  | 514 | 519 | DDFDTL |
| Q92973-2 | TNPO1_HUMAN | Transportin-1 | 719 | 724 | PEFISV |
| P60174-1 | TPIS_HUMAN | Triosephosphate isomerase | 89 | 94 | ATWVVL |
|  |  |  | 239 | 244 | PEFVDI |
| Q9BQE3 | TBA1C_HUMAN | Tubulin alpha-1C chain | 65 | 70 | AVFVDL |
|  |  |  | 147 | 152 | SGFTSL |
|  |  |  | 430 | 435 | KDYEEV |
| P49411 | EFTU_HUMAN | Elongation factor Tu, mitochondrial | 244 | 249 | DTYIPV |
| Q16881-7 | TRXR1_HUMAN | Thioredoxin reductase 1, cytoplasmic | 160 | 165 | ASYVAL |
|  |  |  | 439 | 444 | EVFTTL |
| Q9NNW7-2 | TRXR2_HUMAN | Thioredoxin reductase 2, mitochondrial | 12 | 17 | RDYDLL |
|  |  |  | 200 | 205 | ASYVAL |
|  |  |  | 281 | 286 | GTFDTV |
| P22314-2 | UBA1_HUMAN | Ubiquitin-like modifier-activating enzyme 1 | 117 | 122 | SGFQVV |
| Q92900-2 | RENT1_HUMAN | Regulator of nonsense transcripts 1 | 834 | 839 | KDFIIL |
| P31930 | QCR1_HUMAN | Cytochrome b-c1 complex subunit 1, mitochondrial | 72 | 77 | GVWIDV |
| P22695 | QCR2_HUMAN | Cytochrome b-c1 complex subunit 2, mitochondrial | 38 | 43 | LEFTKL |
| P18206-2 | VINC_HUMAN | Vinculin | 142 | 147 | LEYLTV |
| P08670 | VIME_HUMAN | Vimentin | 381 | 386 | REYQDL |
| O14980 | XPO1_HUMAN | Exportin-1 | 586 | 591 | DTFIKI |
| P13010 | XRCC5_HUMAN | X-ray repair cross-complementing protein 5 | 86 | 91 | PDFDLL |
|  |  |  | 274 | 279 | KTWTVV |
| P12956-2 | XRCC6_HUMAN | X-ray repair cross-complementing protein 6 | 389 | 394 | PGFQLV |
| P54577 | SYYC_HUMAN | Tyrosine-tRNA ligase, cytoplasmic | 174 | 179 | EEYLKV |
|  |  |  | 273 | 278 | SEFVIL |
|  |  |  | 486 | 491 | KVFEKL |
